# Efficacy and safety of pharmacotherapy for recurrent high-grade glioma: a systematic review and network meta-analysis

**DOI:** 10.3389/fphar.2023.1191480

**Published:** 2023-06-01

**Authors:** Yanan Xu, Haijing Guan, Kefu Yu, Nan Ji, Zhigang Zhao

**Affiliations:** ^1^ Department of Pharmacy, Beijing Tiantan Hospital, Capital Medical University, Beijing, China; ^2^ School of Pharmacy, Capital Medical University, Beijing, China; ^3^ Department of Neurosurgery, Beijing Tiantan Hospital, Capital Medical University, Beijing, China

**Keywords:** high-grade glioma, recurrent, pharmacotherapy, network meta-analysis, systematic review, efficacy, safety

## Abstract

**Objective:** To compare the efficacy and safety of treatments for patients with recurrent high-grade gliomas.

**Methods:** Electronic databases including Pubmed, Embase, Cochrane Library and ClinicalTrials.gov were searched for randomized controlled trials (RCT) related to high-grade gliomas. The inclusion of qualified literature and extraction of data were conducted by two independent reviewers. The primary clinical outcome measures of network meta-analysis were overall survival (OS) while progression-free survival (PFS), objective response rate (ORR) and adverse event of grade 3 or higher were secondary measures.

**Results:** 22 eligible trials were included in the systematic review, involving 3423 patients and 30 treatment regimens. Network meta-analysis included 11 treatments of 10 trials for OS and PFS, 10 treatments of 8 trials for ORR, and 8 treatments of 7 trials for adverse event grade 3 or higher. Regorafenib showed significant benefits in terms of OS in paired comparison with several treatments such as bevacizumab (hazard ratio (HR), 0.39; 95% confidence interval (CI), 0.21–0.73), bevacizumab plus carboplatin (HR, 0.33; 95%CI, 0.16–0.68), bevacizumab plus dasatinib (HR, 0.44; 95%CI, 0.21–0.93), bevacizumab plus irinotecan (HR, 0.4; 95%CI, 0.21–0.74), bevacizumab plus lomustine (90 mg/m^2^) (HR, 0.53; 95%CI, 0.33–0.84), bevacizumab plus lomustine (110 mg/m^2^) (HR, 0.21; 95%CI, 0.06–0.7), bevacizumab plus vorinostat (HR, 0.42; 95%CI, 0.18–0.99), lomustine (HR, 0.5; 95%CI, 0.33–0.76), and nivolumab (HR, 0.38; 95%CI, 0.19–0.73). For PFS, only the hazard ratio between bevacizumab plus vorinostat and bevacizumab plus lomustine (90 mg/m^2^) was significant (HR,0.51; 95%CI, 0.27–0.95). Lomustine and nivolumab conferred worse ORR. Safety analysis showed fotemustine as the best and bevacizumab plus temozolomide as the worst.

**Conclusion:** The results suggested that regorafenib and bevacizumab plus lomustine (90 mg/m^2^) provide improvements in terms of survival but may have poor ORR in patients with recurrent high-grade glioma.

## Introduction

According to the latest criteria of World Health Organization classification in 2021 (Louis et al., 2021), high-grade gliomas encompass various types, including grade 3 and 4 astrocytoma, grade 3 oligodendroglioma, and grade 4 glioblastomas (GBM), with GBM being the most common. Despite the fact that high-grade gliomas account for approximately 25% of all brain tumors, they are characterized by high aggression and malignancy, with an inevitable tendency for recurrence (Ostrom et al., 2018). The median progression-free survival (PFS) after recurrence is only 1.8 months (McKinnon et al., 2021), and the median overall survival (OS) ranges between 7.1 and 9.8 months, with a 5-year survival rate of only about 5% (Ostrom et al., 2018).

Surgical resection remains a viable option for treating recurrent high-grade gliomas, particularly in the case of symptomatic or large lesions. Nonetheless, successful outcomes are largely dependent on complete resection (Wen et al., 2020). Due to the extensive and invasive nature of tumor tissue, often infiltrating into healthy surrounding tissue, the success rate of re-operation is limited by factors such as tumor location and structural complexities (Ma et al., 2021).

In cases where radiotherapy is repeated, careful consideration must be given to variables such as the initial radiation dose, time interval since treatment, and the location and volume of the recurrent tumor (Cabrera et al., 2016). However, there are few randomized trials to definitively prove whether radiotherapy prolongs survival time (Wen et al., 2020).

Alternatively, drug therapies have relatively fewer limitations and are often the primary choice for relapsed patients. The drugs currently available for high-grade glioma include bevacizumab, lomustine, temozolomide, regorafenib, PCV (procarbazine, lomustine, and vincristine), and relative drug combinations. However, the clinical benefit of these therapies is limited, as evidenced by the results of numerous clinical trials (Nabors et al., 2020; Weller et al., 2021). With the abundance of clinical trials with inconclusive results (Omuro and DeAngelis, 2013), it becomes perplexing for clinicians to make informed decisions. Therefore, performing a network meta-analysis that compares treatments from varying clinical trials becomes pivotal.

An analysis focusing on recurrent GBM has been previously conducted, (McBain et al., 2021), while it lacked a collection of evidence on grade 3 glioma treatment. Furthermore, fresh clinical study outcomes have emerged that necessitate evaluation. Hence, we performed this systematic review and Bayesian network meta-analysis to amass and summarize the treatment evidence for both grade 3 and 4 gliomas. Additionally, we reconstruct data from published Kaplan-Meier survival curves to include as much clinical evidence as possible and enable comprehensive results. The results of direct and indirect comparisons were integrated to evaluate the efficacy and safety of various drug therapies. We also ranked the clinical measures of each therapeutic regimen to provide a comprehensive assessment for clinical decision-making and to improve prognosis for patients experiencing tumor recurrence.

## Methods

This study was conducted in accordance with Preferred Reporting Items for Systematic Reviews and Meta-analyses (PRISMA) statement (Supplementary Table S1) (Page et al., 2021). The protocol was registered with the international prospective register of systematic reviews (PROSPERO CRD42022383881).

### Data sources and search strategy

A thorough search of PubMed, Embase, Cochrane Central Register, China National Knowledge Infrastructure, WanFang Data Knowledge Service Platform and China Science and Technology Journal Database was conducted for published randomized controlled trials (RCTs) with the inclusion of an additional search of ClinicalTrials.gov for unpublished RCTs. The search terms included “high-grade gliomas,” “anaplastic astrocytoma,” “glioblastoma,” “anaplastic oligoastrocytoma,” “recurren*,“ “relapse,” and drug names. Details of the literature search strategy can be found in Supplementary Table S2, with the search results collected up until 3 August 2022. The listing status of drugs was confirmed through the U.S. Food and Drug Administration (FDA) and drug-approval agencies in other countries.

### Selection criteria

RCTs were included based on the following criteria:

1) Adult patients (≥18 years) with histologically confirmed recurrent high-grade gliomas, including GBM and anaplastic gliomas.
2) Trials that compared two or more arms of drug therapies, such as chemotherapy, immunotherapy and targeted therapy.
3) Trials that reported at least one of the following outcomes:
  (i) OS, defined as the time from randomization to death;
  (ii) PFS, defined as the time from randomization to first progression (local or distant) or death;
  (iii) Objective response rate (ORR), defined as the proportion of patients achieving an objective response;
  (iv) The incidence of grade 3 or higher adverse events (AE), determined according to the common terminology criteria for adverse events.

Duplicate studies and trials that were terminated or closed, along with trials in which drugs had not been approved for marketing by any nation were excluded. Furthermore, study arms that included operation or radiotherapy were disallowed.

Xu and Guan independently excluded irrelevant results by screening titles and abstracts, and included eligible articles by browsing through full texts. Any divergences during selection were resolved through arbitration by all reviewers.

### Data extraction and quality evaluation

The details of the included articles were extracted to a pre-designed form, including publication information (title, first author, year of publication, journal of publication, country, etc.), trial information (trial start and cut-off time, disease, patient inclusion criteria, number of enrolled patients, baseline characteristics of the population, follow-up time), treatment regimens, and outcomes. If the OS and PFS were incomplete, missing data were estimated based on Kaplan-Meier curves following the methods provided by Tierney et al. (2007).

The Cochrane Risk of Bias 2 tool (Cumpston et al., 2019) assessed the individual study’s risk of bias in five areas: randomization process, deviations from intended interventions, missing outcome data, measurement of the outcome and selection of the reported result. Trials were categorized as low risk, high risk, or unclear concern of bias based on the above criteria.

Data extraction was conducted by Xu and Guan, and quality evaluation was conducted independently by Xu and Yu. Any discrepancies that emerged during the evaluation process were resolved through consensus among all reviewers.

### Data synthesis and statistical analysis

The primary study outcome in this study was OS, with secondary outcomes being PFS, ORR, and grade 3 or higher AE. Survival data were presented as the hazard ratio (HR) with corresponding 95% confidence interval (CI), while categorical variables were expressed as the odds ratio (OR) with corresponding 95% CI.

Bayesian network meta-analysis was conducted due to its adaptability with complicated situations and its ability to explain the effects of study-specific covariates, leading to accurate estimates with limited information. Additionally, it provides a straightforward approach to carry out probabilistic statements and treatment effect predictions (Salanti et al., 2011). Network diagrams were generated for different treatment outcomes using Stata (version 17) (Chaimani et al., 2013). Fixed-effects and random-effects models were established separately through a Markov Chain Monte Carlo simulation technique in R (version 4.2.2) with 150000 iterations, 30000 burn-ins and a thinning interval of 1, based on the Bayesian framework (Salanti et al., 2011). The final appropriate analytical model was chosen based on the model parameters. Convergence was assessed through visual inspection of trace plots, density plots and Brooks-Gelman Rubin diagnosis plots (Supplementary Figure S1). Heterogeneity was evaluated using I^2^ statistics, with values categorized as low, medium and high heterogeneity for I^2^ values < 25%, 25%–50% and >50%, respectively, (Higgins et al., 2003). Global consistency was assessed by comparing the consistent and inconsistent models (Dias et al., 2010). The inconsistency of the models was assessed using the node splitting method (Higgins et al., 2003). Probability plots and surface under the cumulative ranking curve (SUCRA) were used to predict and evaluate the efficacy and safety of each treatment.

A sensitivity analysis was conducted to evaluate the reliability and stability of network meta-analysis results, with articles causing greater heterogeneity excluded for sensitive analysis.

## Results

### Systematic review and characteristics

**FIGURE 1:**
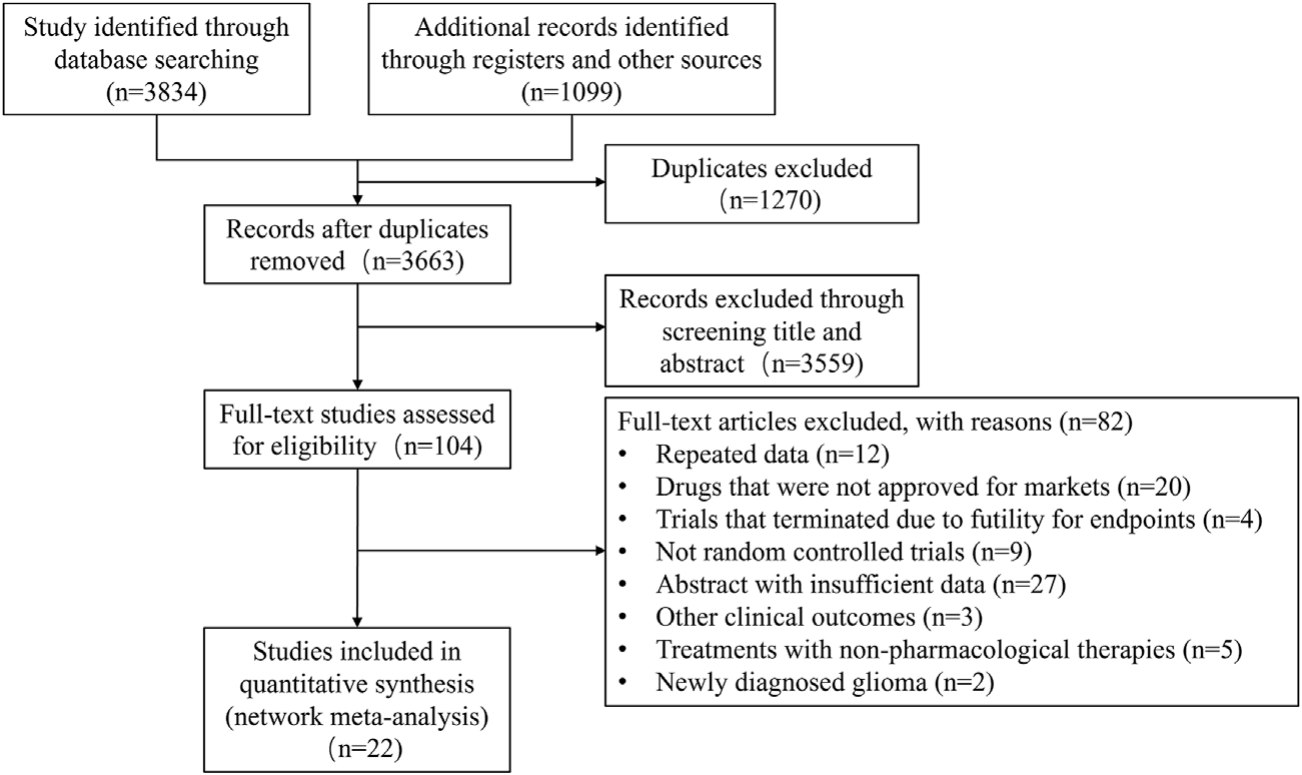
Study selection.

In this study, a total of 104 out of 4933 records for full-text reading and 22 RCTs (Boiardi et al., 1992; Yung et al., 2000; Friedman et al., 2009; Brada et al., 2010; Dresemann et al., 2010; Song et al., 2010; Sun et al., 2013; Taal et al., 2014; Field et al., 2015; Reardon et al., 2015; Brandes et al., 2016; Gilbert et al., 2017; Wick et al., 2017; Duerinck et al., 2018; Brandes et al., 2019; Galanis et al., 2019; Lombardi et al., 2019; Puduvalli et al., 2020; Reardon et al., 2020; Nayak et al., 2021; Twelves et al., 2021; Patil et al., 2022) were included for analysis (Figure 1). The study population consisted of 3423 patients who received 30 different treatments. Bevacizumab, lomustine and temozolomide were the most commonly studied. The characteristics of the tumor types, number of tumor recurrences, sex ratio, age, Karnofsky performance status (KPS), and specific treatment regimens were summarized in Table 1. The risk of bias assessment in the literature was evaluated and presented in Supplementary Figure S2.

**TABLE 1:** Baseline characteristic of included studies of patients with high-grade glioma.

Study id	Tumor types	Number of relapses	Number of patients	Female (%)	Median age	KPS ≥ 80 (%)	Regimens	Reported outcomes
Boiardi et al, 1992	GBM	NM	19	NM	56	NM	Vincristine 2 mg; lomustine 75 mg/m^2^; procarbazine75 mg/m^2^;hydroxyurea 1500 mg/m^2^; cisplatin 90 mg/m^2^; algocytidine 300 mg/m^2^; dacarbazine 150 mg/m^2^ and methylprednisolone 300 mg/m^2^ were administered every 6 h for 3 does.	ORR
			16		61	NM	Lomustine 110 mg/m^2^ was administered on day 1, procarbazine 60 mg/m^2^ was administered daily for 14 days beginning on day 8, and vincristine 1.4 mg/m^2^ was administered on day 8 and 29 of each 6 weeks cycle of therapy.	
Brada et al, 2010	AA, GBM, gliosarcoma, oligoastrocytoma, gliosarcoma	1	112	35.7	53	NM	TMZ 200 mg/m^2^ on day 1–5 every 28 days.	OS, PFS, Grade ≥3 AEs
			111	36.9	53	NM	TMZ 100 mg/m^2^ on day 1–21 every 28 days.	
			224	34.8	53	NM	lomustine 110 mg/m^2^ on day 1, procarbazine 60 mg/m^2^ once a day on day 8–21 and vincristine 1.4 mg/m^2^ on day 8 and 29 every 6 weeks.	
Brandes et al, 2016	GBM	1	32	28.1	56	NM	Fotemustine 75 mg/m^2^ on days 1, 8, and 15. After a 35-day break, fotemustine 100 mg/m^2^ every 3 weeks.	OS, PFS, Grade ≥3 AEs
			59	33.9	59	NM	Bevacizumab 10 mg/kg every 2 weeks.	
Brandes et al, 2019	GBM	1	61	27.9	56	90	Lomustine 90 mg/m^2^ every 6 weeks. Bevacizumab 10 mg/kg every 2 weeks.	OS, PFS, Grade ≥3 AEs
			62	27.4	58.5	92	Lomustine 110 mg/m^2^ every 6 weeks.	
Dresemann et al, 2010	GBM	1, 2	120	41.7	52	NM	Imatinib 600 mg once a day. Hydroxyurea 500 mg twice a day.	OS, PFS, Grade ≥3 AEs
			120	31.7	51	NM	Hydroxyurea 500 mg 3 times a day.	
Duerinck et al, 2018	GBM	1, 2	29	37.9	56	NM	Axitinib 5 mg twice a day. Lomustine 90 mg/m^2^ every 6 weeks.	OS, PFS, ORR, Grade ≥3 AEs
			50	34.0	55	NM	Axitinib 5 mg twice a day.	
Field et al, 2015	GBM	1, 2	60	43.3	55	82	Carboplatin AUC 5 every 4 weeks. Bevacizumab 10 mg/kg every 2 weeks.	OS, PFS, ORR, Grade ≥3 AEs
			62	46.8	55	84	Bevacizumab 10 mg/kg every 2 weeks.	
Friedman et al, 2009	GBM	1, 2	82	30.5	57	100	Irinotecan 125 mg/m^2^ every 2 weeks. Bevacizumab 10 mg/kg every 2 weeks.	OS, PFS, ORR, Grade ≥3 AEs
			85	31.8	54	100	Bevacizumab 10 mg/kg every 2 weeks	
Galanis et al, 2019	GBM	NM	83	33.7	58	NM	Dasatinib 100 mg twice a day. Bevacizumab 10 mg/kg every 2 weeks.	OS, PFS, ORR, Grade ≥3 AEs
			38	42.1	56.5	NM	Bevacizumab 10 mg/kg every 2 weeks.	
Gilbert et al, 2017	GBM or Gliosarcoma	NM	60	43.3	58	100	TMZ 75 mg/m^2^ on day 1–21 every 28 days. Bevacizumab 10 mg/kg every 2 weeks.	OS, PFS, ORR, Grade ≥3 AEs
			57	40.4	55	100	Irinotecan 125 mg/m^2^ every 2 weeks. Bevacizumab 10 mg/kg every 2 weeks.	
Lombardi et al, 2019	GBM	1	59	30.5	54.8	NM	Regorafenib 160 mg once a day for the first 3 weeks of each 4-week.	OS, PFS, ORR, Grade ≥3 AEs
			60	28.3	58.9	NM	Lomustine 110 mg/m^2^ every 6 weeks.	
Nayak et al, 2021	GBM	1, 2	50	30.0	52	100	Pembrolizumab 200 mg every 3 weeks. Bevacizumab 10 mg/kg every 2 weeks.	OS, PFS, ORR
			30	36.7	55	100	Pembrolizumab 200 mg every 3 weeks.	
Patil et al, 2022	GBM	NM	44	25.0	40.5	NM	Mebendazole 1600 mg 3 times a day. TMZ 200 mg/m^2^ on day 1–5 every 28 days.	OS, PFS, Grade ≥3 AEs
			44	27.3	41	NM	Mebendazole 800 mg 3 times a day. Lomustine 110 mg/m^2^ on day 1 every 6 weeks.	
Puduvalli et al, 2020	Grade IV glioma	1, 2, 3	47	36.2	NM	94	Vorinostat 400 mg on day 1–7 and 15–21 every 4 weeks. Bevacizumab 10 mg/kg every 2 weeks.	OS, PFS, Grade ≥3 AEs
			38	26.3		97	Bevacizumab 10 mg/kg every 2 weeks.	
Reardon et al, 2015	Grade IV glioma	1	41	34.1	56.6	100	Afatinib 40 mg once a day.	OS, PFS, ORR, Grade ≥3 AEs
			39	46.2	55.4	100	Afatinib 40 mg once a day. TMZ 75 mg/m^2^ on day 1–21 every 28 days.	
			39	35.9	56.9	100	TMZ 75 mg/m^2^ on day 1–21 every 28 days.	
Reardon et al, 2020	GBM or Gliosarcoma	1	184	37.0	55.5	99	Nivolumab 3 mg/kg every 2 weeks.	OS, PFS, ORR, Grade ≥3 AEs
			185	35.7	55	100	Bevacizumab 10 mg/kg every 2 weeks.	
Song et al, 2010	GBM	NM	23	NM	NM	NM	Hydroxycamptothecin 6 mg/m^2^ on day 1–7 every 28 days.	OS, PFS, ORR
			24	NM	NM	NM	TMZ 150 mg/m^2^ on day 1–5 every 28 days.	
Sun et al, 2013	GBM	NM	65	40.0	45.1	NM	Semustine 150 mg/m^2^ on day 1 every 28 days.	ORR
			79	30.4	44.3	NM	TMZ 150 or 200 mg/m^2^ on day 1–5 every 28 days.	
Taal et al, 2014	GBM	1	50	38.0	58	NM	Bevacizumab 10 mg/kg every 2 weeks.	OS, PFS, ORR
			46	43.5	56	NM	Lomustine 110 mg/m^2^ every 6 weeks.	
			8	62.5	53	NM	Lomustine 110 mg/m^2^ every 6 weeks. Bevacizumab 10 mg/kg every 2 weeks.	
			44	31.8	58	NM	Lomustine 90 mg/m^2^ every 6 weeks. Bevacizumab 10 mg/kg every 2 weeks.	
Twelves et al, 2021	GBM	1	12	58.3	59	91	TMZ 85 mg/m^2^ on day 1–21 every 28 days. Nabiximols 3–12 sprays daily.	Grade ≥3 AEs
			9	11.1	57	100	TMZ 85 mg/m^2^ on day 1–21 every 28 days.	
Wick et al, 2017	GBM	1	149	38.9	59.8	NM	Lomustine 110 mg/m^2^ every 6 weeks.	OS, PFS, ORR, Grade ≥3 AEs
			288	39.6	57.1	NM	Lomustine 90 mg/m^2^ every 6 weeks. Bevacizumab 10 mg/kg every 2 weeks.	
Yung et al, 2000	GBM or Gliosarcoma	1	112	31.3	52	100	TMZ 150 or 200 mg/m^2^ on day 1–5 every 28 days.	OS, PFS, ORR, Grade ≥3 AEs
			113	36.3	52	99	Procarbazine 125 or 150 mg/m^2^ on day 1–28 every 56 days.	

AA, anaplastic astrocytoma; GBM, glioblastoma; NM, not mentioned; ORR, objective response rate; OS, overall survival; PFS, progression-free survival; TMZ, temozolomide.

### Network meta-analysis

#### Network evidence plots

**FIGURE 2:**
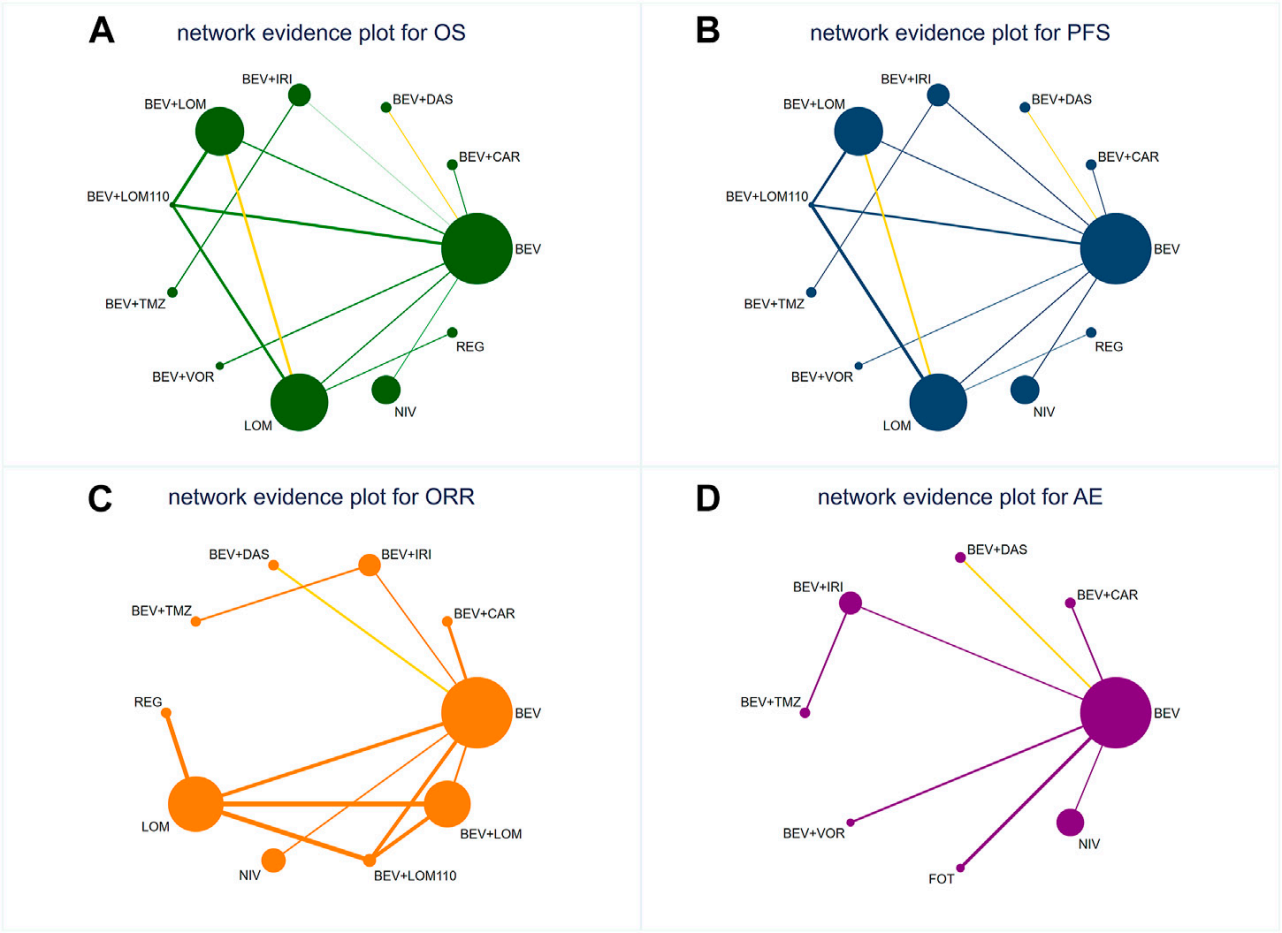
Network diagrams of comparisons on different outcomes of treatments s in different groups for patients with recurrent high-grade glioma. The yellow line indicates that there are studies in this comparison group that implemented a blinded approach. **(A)** Comparison of network diagrams for OS in high-grade glioma. **(B)** Comparison of network diagrams for PFS in high-grade glioma. **(C)** Comparison of network diagrams for ORR in high-grade glioma. **(D)** Comparison of network diagrams for grade 3 or higher AEs in high-grade glioma. BEV, bevacizumab; CAR, carboplatin; DAS, dasatinib; IRI, irinotecan; LOM, lomustine (90 mg/m^2^); LOM110, lomustine (110 mg/m^2^); TMZ, temozolomide; VOR, vorinostat; NIV, nivolumab; REG, regorafenib; FOT, fotemustine.

A network meta-analysis was conducted to assess the efficacy and safety of the different treatment regimens. A total of 11 treatment regimens from 10 studies (Friedman et al., 2009; Taal et al., 2014; Field et al., 2015; Gilbert et al., 2017; Wick et al., 2017; Brandes et al., 2019; Galanis et al., 2019; Lombardi et al., 2019; Puduvalli et al., 2020; Reardon et al., 2020) constituted the analysis network for OS and PFS (Figures 2A, B), and 10 treatment regimens from 8 studies (Friedman et al., 2009; Taal et al., 2014; Field et al., 2015; Gilbert et al., 2017; Wick et al., 2017; Galanis et al., 2019; Lombardi et al., 2019; Reardon et al., 2020) constituted the analysis network for ORR (Figure 2C). Furthermore, 8 treatment regimens from 7 studies (Friedman et al., 2009; Field et al., 2015; Brandes et al., 2016; Gilbert et al., 2017; Galanis et al., 2019; Puduvalli et al., 2020; Reardon et al., 2020) constituted the analysis network for AEs (Figure 2D).

#### Heterogeneity and inconsistency assessment

A fixed-effects model was used for the analysis of OS, ORR and AEs (I^2^ < 25%) and a random-effects model for the analysis of PFS (I^2^ > 50%). The results of heterogeneity test were presented in Supplementary Figure S3. The heterogeneity of the comparison group of lomustine and bevacizumab plus lomustine was high (I^2^ = 69.6%), which was mainly due to the Brandes2019 study. After removing this trial, the I^2^ decreased to 32.4% (Supplementary Figure S3E). A closed-loop structure was present in the network of OS, PFS, and ORR, but since the arms that comprised the loop were from one literature, (Taal et al., 2014), there was no need to check the consistency of the direct evidence (van Valkenhoef et al., 2016).

#### Comparison of efficacy and safety

The direct and indirect evidence of different treatments in terms of survival and binary outcomes were synthesized and reported as HR and OR, respectively.

**FIGURE 3:**
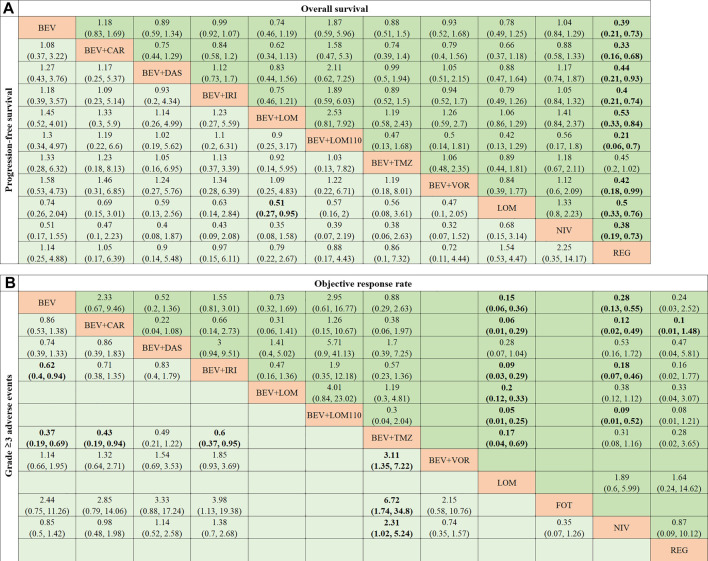
Pooled estimates of the network meta-analysis. **(A)** Pooled HRs (95% credible intervals) for OS in the upper triangle and PFS in the lower triangle. **(B)** Pooled ORs (95% credible intervals) for ORR in the upper triangle and 3 or higher AEs in the lower triangle. BEV, bevacizumab; CAR, carboplatin; DAS, dasatinib; IRI, irinotecan; LOM, lomustine (90 mg/m^2^); LOM110, lomustine (110 mg/m^2^); TMZ, temozolomide; VOR, vorinostat; NIV, nivolumab; REG, regorafenib; FOT, fotemustine.

Regorafenib was found to have the best benefit for OS (Figure 3A), compared to other treatment regimens. In terms of PFS (Figure 3A), only bevacizumab plus lomustine (90 mg/m^2^) was significantly effective than lomustine alone (HR = 0.51, 95% CI 0.27-0.95). The HR of bevacizumab plus lomustine (90 mg/m^2^) was less than 1, which had a therapeutic advantage compared to the other nine regimens, though the confidence interval spanned 1.

Lomustine and nivolumab performed poorly on ORR (Figure 3B). The range of ORs were from 0.05 to 0.28 for lomustine compared to bevacizumab, bevacizumab plus carboplatin, bevacizumab plus dasatinib, bevacizumab plus irinotecan, bevacizumab plus lomustine (including 90 mg/m^2^ and 110 mg/m^2^) and bevacizumab plus temozolomide. The range of ORs were from 0.09 to 0.53 for nivolumab compared to the above regimens.

For grade 3 or higher AE (Figure 3B), bevacizumab plus temozolomide suffered the worst safety. The primary adverse event of bevacizumab plus temozolomide was myelotoxicity (Gilbert et al., 2017).

**FIGURE 4:**
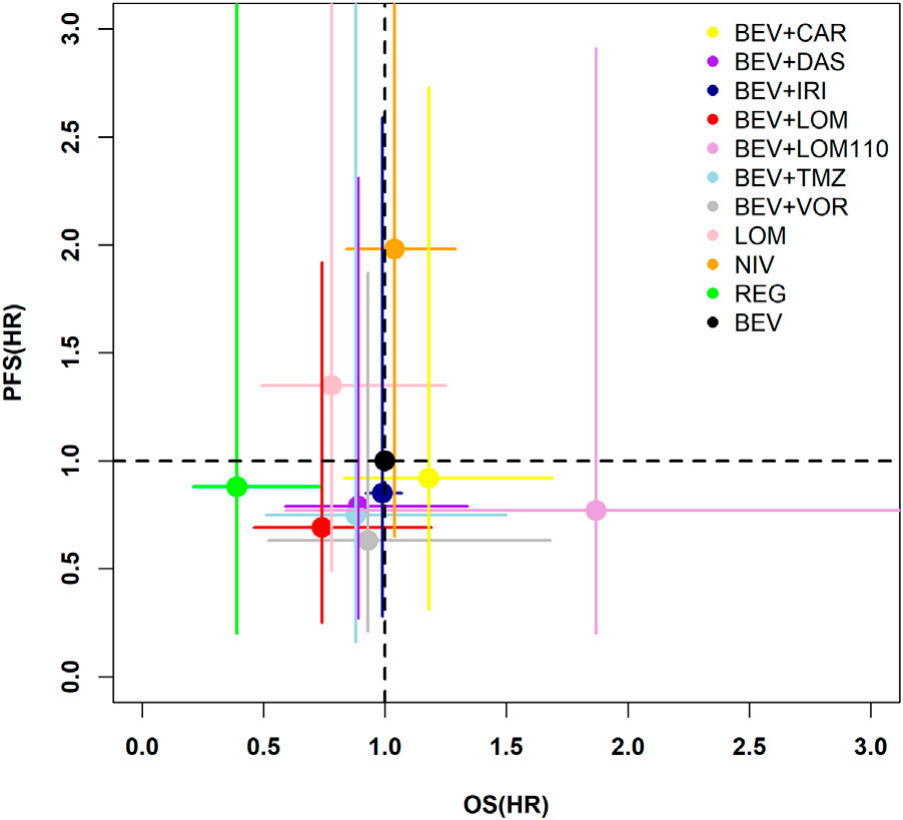
Two-dimensional plot of OS and PFS for difference treatments. The horizontal coordinate indicates the risk ratio for OS of the study regimen with bevacizumab as the control, and the vertical coordinate indicates the risk ratio for PFS of the study regimen with bevacizumab as the control. The dots indicate the estimated risk ratios for the study regimens, and the horizontal line indicates the 95% confidence interval for HR. BEV, bevacizumab; CAR, carboplatin; DAS, dasatinib; IRI, irinotecan; LOM, lomustine (90 mg/m^2^); LOM110, lomustine (110 mg/m^2^); TMZ, temozolomide; VOR, vorinostat; NIV, nivolumab; REG, regorafenib.

A two-dimensional graph was drawn to visualize the effect of different treatments on OS and PFS, taking bevacizumab as control (Figure 4). The diagram showed that regorafenib, bevacizumab plus lomustine (90 mg/m^2^), bevacizumab plus temozolomide, bevacizumab plus dasatinib, and bevacizumab plus vorinostat had better efficacy than bevacizumab in terms of OS and PFS, athough the confidence interval for HR of most regimens crossed 1 with no significant difference.

#### Rank probabilities

**FIGURE 5:**
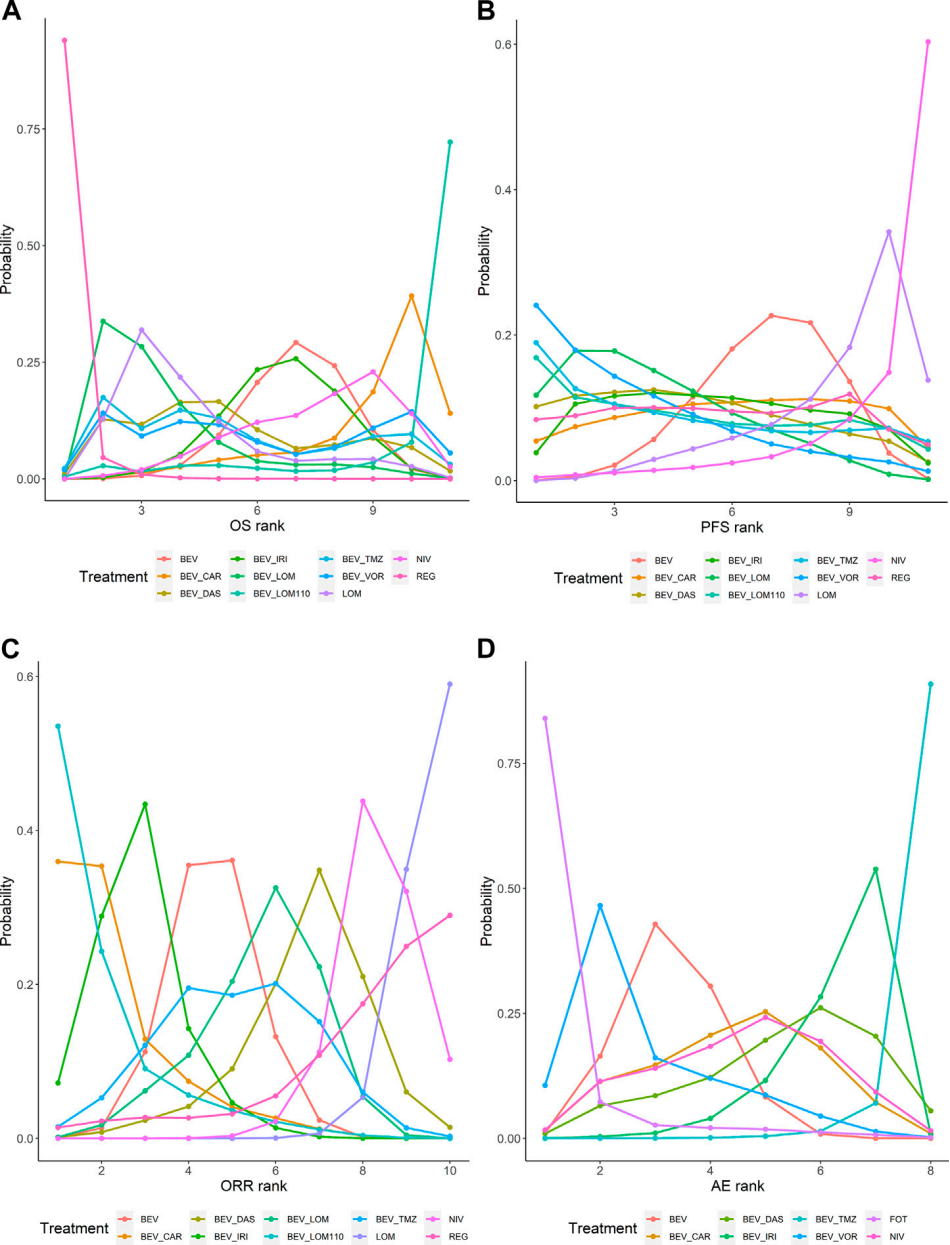
Bayesian ranking profiles of comparable treatments on efficacy and safety for patients with high-grade gliomas. Profiles indicate the probability of each treatment being ranked from first to last on OS **(A)**, PFS **(B)**, ORR **(C)**, and grade 3 or higher AEs **(D)**. Ranking curves are described according to the Bayesian ranking results presented in Supplementary Table S3. BEV, bevacizumab; CAR, carboplatin; DAS, dasatinib; IRI, irinotecan; LOM, lomustine (90 mg/m^2^); LOM110, lomustine (110 mg/m^2^); TMZ, temozolomide; VOR, vorinostat; NIV, nivolumab; REG, regorafenib; FOT, fotemustine.

**FIGURE 6:**
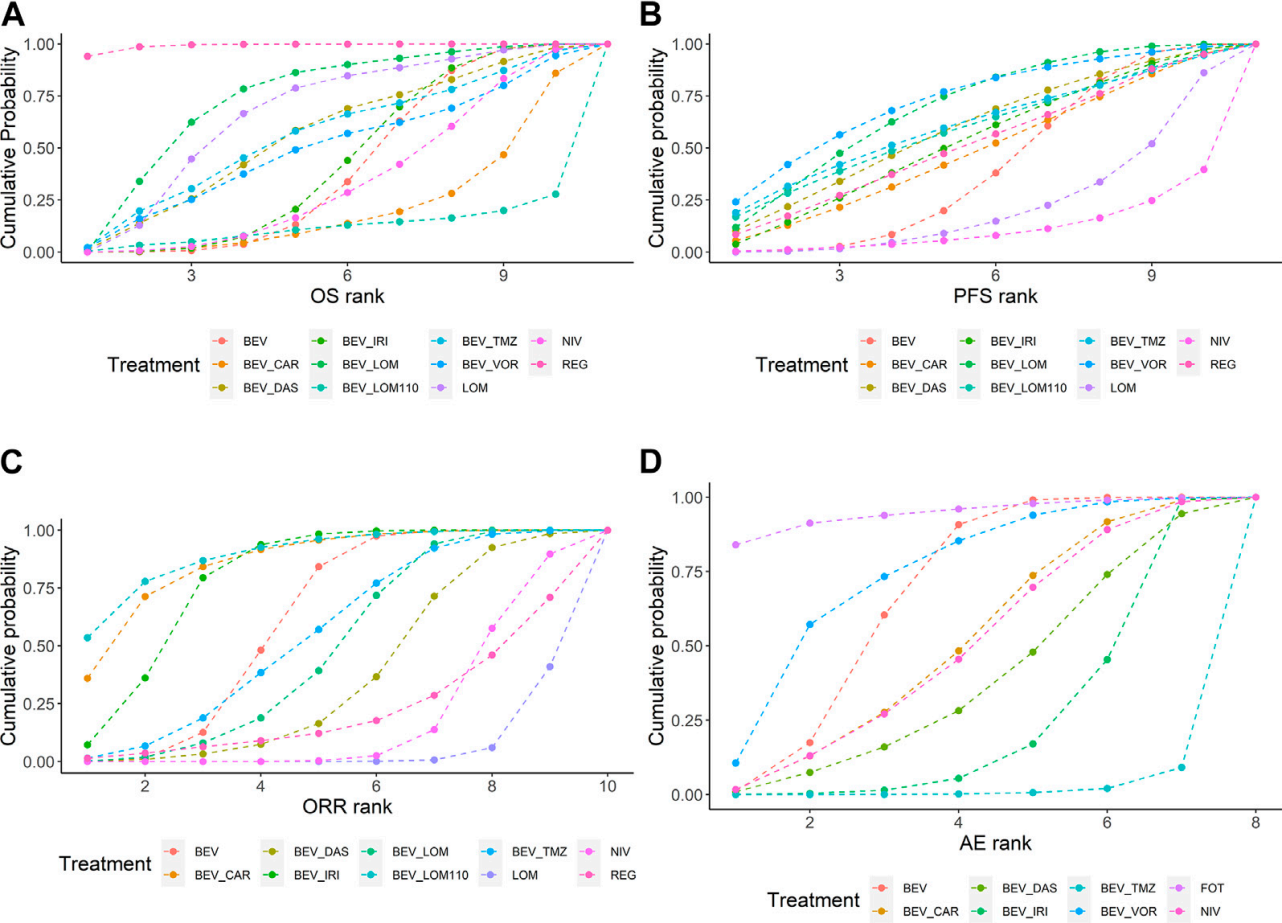
SUCRA ranking of comparable treatments on efficacy and safety for patients with high-grade gliomas. Profiles indicate the cumulative probability of each treatment being ranked in the top on OS **(A)**, PFS **(B)**, ORR **(C)**, and grade 3 or higher AEs **(D)**. SUCRA are described according to the Bayesian cumulative ranking results presented in Supplementary Table S4. BEV, bevacizumab; CAR, carboplatin; DAS, dasatinib; IRI, irinotecan; LOM, lomustine (90 mg/m^2^); LOM110, lomustine (110 mg/m^2^); TMZ, temozolomide; VOR, vorinostat; NIV, nivolumab; REG, regorafenib; FOT, fotemustine.

The ranking and SUCRA of comparable treatments for patients with high-grade glioma obtained by network meta‐analysis (Figures 5, 6) were consistent with HR and OR.

Patients with recurrent high-grade glioma treated with regorafenib are likely to experience the longest OS (94% probability). The SUCRA of regorafenib was much higher than other regimens. Patients treated with bevacizumab plus vorinostat may attain the longest PFS (24% probability). However, the SUCRA of bevacizumab plus lomustine (90 mg/m^2^) and bevacizumab plus vorinostat were similar. Patients treated with bevacizumab plus lomustine (110 mg/m^2^) may have better ORRs (54% probability). Lomustine and nivolumab performed poorly for ORR. Patients treated with lomustine were minimally at risk for a grade ≥3 AEs (84% probability), whereas bevacizumab-based regimens tended to have higher toxicity than bevacizumab alone.

#### Sensitivity analysis

There was a large heterogeneity of PFS after combining various trials. Therefore, a sensitivity analysis was conducted, excluding each trial in turn. As a result, it was identified that Brandes2019 was the primary source of heterogeneity in the PFS network. With this information in mind, sensitivity analyses of PFS outcomes were performed using the remaining studies, excluding Brandes 2019. Supplementary Figure S4 display the results of pairwise comparison, probability ranking, and the SUCRA.

The sensitivity analysis outcomes aligned with those yielded by the Bayesian network meta-analysis. In the pairwise comparison, it remained that bevacizumab plus lomustine (90 mg/m^2^) achieved a significantly enhanced PFS, as compared to lomustine. The HRs of other comparisons were not found to be significant.

Similarly, in the ranking of PFS, the curves followed the same pattern as the network meta-analysis. Additionally, there was a slight increase in the probability that bevacizumab plus lomustine (90 mg/m^2^) would rank in the top four. Global results obtained from the network meta-analysis were robust.

## Discussion

In this systematic review and Bayesian network meta-analysis, we present a comprehensive summary and comparison of the efficacy and safety profiles of various interventions for high-grade gliomas, including bevacizumab monotherapy, bevacizumab-based therapies, nitrosoureas, PD-1 inhibitors, and multi-targeted kinase inhibitors. To bolster the study’s clinical utility in real-world practice, we excluded investigations on experimental drugs that remain unavailable commercially.

The results of the study suggest that regorafenib is likely to be the most effective treatment for improving survival outcomes in patients with longer OS and PFS, although the options may not provide the same benefit in terms of ORR. The efficacy of the combination therapy of bevacizumab and lomustine (90 mg/m^2^) was inferior to that of regorafenib. Nevertheless, it outperformed other treatment options in terms of survival outcomes and is recommended as the second option according to our findings. However, the ORR was unsatisfactory as well. Notably, bevacizumab plus lomustine (110 mg/m^2^) ranked high in ORR outcomes, which may be attributed to the dose administered. However, the limited sample size of patients in original studies may have introduced some bias into our results.

In terms of safety, no drug had an absolutely good safety profile. Regorafenib and bevacizumab plus lomustine (90 mg/m^2^) were not included in the safety evaluation network due to incomplete safety data. Grade 3 or higher AEs for regorafenib were dominated by elevated lipase and hand-foot skin reactions (both incidences were over 10%) (Lombardi et al., 2019). Main AEs for bevacizumab plus lomustine (90 mg/m2) were hypertension, hematologic effects, and fatigue (Taal et al., 2014; Wick et al., 2017). Our investigation revealed that fotemustine exhibited the most favorable safety profile. Given that it belongs to the same class of nitrosourea as lomustine, it appears reasonable to assert that nitrosourea drugs may generally confer a measure of therapeutic advantage in terms of safety.

The study by Brandes in 2019 exhibited a greater degree of heterogeneity in contrast to the research conducted by Taal in 2014 and Wick in 2017 in paired comparison of lomustine and bevacizumab plus lomustine (90 mg/m^2^). This may be attributed to the ratio of O-6-methylguanine-DNA methyltransferase (MGMT) methylation and unmethylation, which was approximately 1/2 in the Brandes2019 study, compared to a near 1:1 ratio seen in the other two studies. A multitude of investigations have demonstrated an association between MGMT unmethylation and resistance to chemotherapeutic agents (Oldrini et al., 2020). This may plausibly account for the observed larger HR for PFS in the Brandes2019 study, in contrast to the Taal2014 and Wick2017 studies, where the HR values were quite similar. However, we are reassured that our final results were not impacted by heterogeneity through sensitivity analyses.

The study enrolled predominantly patients with recurrent GBM. The National Comprehensive Cancer Network (NCCN) clinical practice guidelines for central nervous system cancers (Nabors et al., 2020) recommend preferential use of bevacizumab, temozolomide, lomustine or carmustine, PCV and regorafenib for recurrent GBM. The European Association of Neuro-Oncology (EANO) guidelines of diffuse glioma in adults (Weller et al., 2021) endorse nitrosoureas, temozolomide and bevacizumab for progression or relapse of GBM. The findings of our analysis support the use of regorafenib for recurrent GBM based on its association with significant survival benefits. However, experience with regorafenib in recurrent GBM is limited compared with other recommended therapeutic options in the guidelines. Regorafenib is a multi-kinase inhibitor. Its anti-tumor mechanism remains elusive despite several clinical trials. A recent investigation delving into its mode of action has unearthed regorafenib’s ability to stabilize the critical enzyme PSAT1 (phosphoserine aminotransferase 1) involved in serine synthesis. This unfavorable activity in GBM cells leads to fatal autophagy arrest and tumor suppression (Jiang et al., 2020). The promising results suggest that the levels of PSAT1 play a key regulatory role in the success of regorafenib-induced GBM therapy. Additional research has identified molecular features correlated with prolonged survival rate in regorafenib-treated GBM patients. These features include EGFR mutations (Chiesa et al., 2022), gene transcripts such as HIF1A and CDKN1A, miRNAs like miR-3607–3p, miR-301a-3p, miR-93–5p (Santangelo et al., 2021), and MAPK pathway mutations that may associate with a poor prognosis (Chiesa et al., 2022). However, limited evidence restricts the scope of individualized dosing of regorafenib, hence, greater evidence is required to increase its widespread acceptance.

The Chinese guidelines (Jiang et al., 2021) recommended bevacizumab plus lomustine (90 mg/m^2^), while it is not a preferred regimen in NCCN(7) and EANO(8) guidelines. Clinicians need to carefully consider the AEs and patient status when selecting this combination. Additionally, cost effectiveness is also an important factor to consider (Cagney and Alexander, 2017), but there is a paucity of evidence in this area at present.

There are commendable aspects to our review, particularly the emphasis placed on high-grade glioma, as opposed to recurrent GBM, although the latter still featured prominently in the final analysis. We established a comprehensive network pertaining to all drug treatments, and judiciously applied analytical methods to estimate hazard ratios founded upon Kaplan-Meier curves, yielding the added benefit of integrating studies that did not report hazard ratios, thus allowing for a more comprehensive evaluation of the many treatments evaluated. Our review presents valuable information for clinical decision-making, which we achieved by carefully scrutinizing and assessing the outcomes of various treatments, and performing rigorous analyses, including sensitivity analysis of network heterogeneity and consistency, thus ensuring robust and dependable results.

Our research, though valuable, still presents some limitations. Firstly, the scope of this study was limited to patients with recurrent high-grade glioma. However, upon examining relevant literature, we discovered a dearth of randomized controlled trials pertaining to grade 3 glioma or anaplastic glioma. Furthermore, we were unable to perform a comprehensive subgroup analysis of grade 3 glioma due to insufficient data. It is thus imperative to acknowledge that the results of our work may not fully represent the ideal treatment strategies for grade 3 recurrent glioma. Another shortcoming of the study was incomplete reporting of results, which prevented the integration of certain guidelines-recommended treatments, such as temozolomide and PCV, into the network. Despite these limitations, pertinent data of clinical trials can be gleaned from Table 1. Language bias may also have some impact on the results. The literature in this article is from English and Chinese databases and may miss potential and qualified studies from other language databases.

As per our research findings, conventional drugs appear to be ineffective in producing significant impacts towards recurrent high-grade glioma. The large molecular phenotype heterogeneity is likely a contributing factor (Nicholson and Fine, 2021). Targeting specific pathways may be a more effective approach (Le Rhun et al., 2019). Among the targeted agents analyzed in this study, both bevacizumab and regorafenib interact with vascular endothelial growth factor (VEGF), which inhibits neoangiogenesis and thus exerts anti-tumor effects. In addition, regorafenib targets multiple gene and kinase such as BRAF, KIT, and RET, which may be potential therapeutic targets but need to be confirmed by further studies. The latest study has found that patients presenting a BRAF-V600E mutation showed improved ORR with dabrafenib and trametinib, providing a clear indication of the potential benefits of individualized treatment strategies (Wen et al., 2022). Likewise, a phase 3 clinical trial of a vaccine has shown promising results in the treatment of recurrent glioma. As demonstrated by Liah et al.’s study, the addition of an autologous tumor lysate-loaded dendritic cell vaccine has resulted in significant clinical benefits resulting in a statistically significant increase in survival time for patients with relapsed GBM (Liau et al., 2023). Whether by targeting specific molecules, pathways, or through autologous tumor lysates, individualized therapy holds significant promise for the treatment of recurrent high-grade gliomas. However, current advancements in this critical area have been insufficient to fully realize the potential of personalized medicine in this setting. In the present context, emerging data emphasizes that regorafenib and bevacizumab in combination with lomustine, represents the most promising therapeutic alternative for high-grade glioma.

## Data Availability

The original contributions presented in the study are included in the article/Supplementary Material, further inquiries can be directed to the corresponding author.
