# Supplementary material for: Efficacy and safety of pharmacotherapy for recurrent high-grade glioma: a systematic review and network meta-analysis
Source: Front Pharmacol. 2023 Jun 1;14:1191480. doi: 10.3389/fphar.2023.1191480 (PMC10267383; doi:10.3389/fphar.2023.1191480)
Supplement: Supplementary file 1 [file DataSheet1.docx]

**Supplementary Material**

**Table of Contents**

|  |  | Page |
| --- | --- | --- |
| **Supplementary Table 1** | Checklist of the PRISMA extension for network meta-analysis | **2-7** |
| **Supplementary Table 2** | Literature search strategies | **8-10** |
| **Supplementary Table 3** | Bayesian ranking results of network meta-analysis | **11,12** |
| **Supplementary Table 4** | Surface under the cumulative ranking curve results of network meta-analysis | **13,14** |
| **Supplementary Figure 1** | Convergence of the three chains established by trace and the Brooks-Gelman-Rubin diagnostic | **15-21** |
| **Supplementary Figure 2** | Results of risk of bias assessment using the Cochrane risk of bias tool 2 | **22** |
| **Supplementary Figure 3** | Forest plots and I2 of head-to-head comparisons | **23-27** |
| **Supplementary Figure 4** | Bayesian ranking results of the sensitivity analysis. | **28, 29** |

**Supplementary Table 1. Checklist of the PRISMA extension for network meta-analysis.**

| **Section/topic** | **#** | **Checklist item*** | **Reported on page #** |
| --- | --- | --- | --- |
| **TITLE** | | |  |
| Title | 1 | Identify the report as a systematic review *incorporating a network meta- analysis (or related form of meta-analysis).* | 1 |
| **ABSTRACT** | | |  |
| Structured summary | 2 | Provide a structured summary including, as applicable:   - Background: main objectives; - Methods: data sources; study eligibility criteria, participants, and interventions; study appraisal and *synthesis methods, such as network meta- analysis.* - Results: number of studies and participants identified; summary estimates with corresponding confidence/credible intervals; *treatment*   *rankings may also be discussed. Authors may choose to summarize pairwise comparisons against a chosen treatment included in their analyses for brevity.*   - Discussion/Conclusions: limitations; conclusions and implications of findings. - Other: primary source of funding; systematic review registration number with registry name. | 1 |
| **INTRODUCTION** | | |  |
| Rationale | 3 | Describe the rationale for the review in the context of what is already known, *including mention of why a network meta-analysis has been conducted.* | 1-2 |
| Objectives | 4 | Provide an explicit statement of questions being addressed with reference to participants, interventions, comparisons, outcomes, and study design (PICOS). | 2 |
| **METHODS** | | |  |
| Protocol and registration | 5 | Indicate if a review protocol exists, if and where it can be accessed (e.g., Web address), and, if available, provide registration information including registration number. | 2 |
| Eligibility criteria | 6 | Specify study characteristics (e.g., PICOS, length of follow-up) and report characteristics (e.g., years considered, language, publication status) used as criteria for eligibility, giving rationale. *Clearly describe eligible treatments included in the treatment network, and note whether any have been clustered or merged into the same node (with justification).* | 3 |
| Information sources | 7 | Describe all information sources (e.g., databases with dates of coverage, contact with study authors to identify additional studies) in the search and date last searched. | 2-3 |
| Search | 8 | Present full electronic search strategy for at least one database, including any limits used, such that it could be repeated. | Supplementary material 8-10 |
| Study selection | 9 | State the process for selecting studies (i.e., screening, eligibility, included in systematic review, and, if applicable, included in the meta-analysis). | 2-4 |
| Data collection process | 10 | Describe method of data extraction from reports (e.g., piloted forms, independently, in duplicate) and any processes for obtaining and confirming data from investigators. | 3 |
| Data items | 11 | List and define all variables for which data were sought (e.g., PICOS, funding sources) and any assumptions and simplifications made. | 2-3 |
| Geometry of the network | S1 | Describe methods used to explore the geometry of the treatment network under study and potential biases related to it. This should include how the evidence base has been graphically summarized for presentation, and what characteristics were compiled and used to describe the evidence base to readers. | 3 |
| Risk of bias in individual studies | 12 | Describe methods used for assessing risk of bias of individual studies (including specification of whether this was done at the study or outcome level), and how this information is to be used in any data synthesis. | 3 |
| Summary measures | 13 | State the principal summary measures (e.g., risk ratio, difference in means). *Also describe the use of additional summary measures assessed, such as treatment rankings and surface under the cumulative ranking curve (SUCRA) values, as well as modified approaches used to present summary findings from meta-analyses.* | 3 |
| Synthesis of results | 14 | Describe the methods of handling data and combining results of studies for each network meta-analysis. This should include, but not be limited to:   - *Handling of multi-arm trials;* - *Selection of variance structure;* - *Selection of prior distributions in Bayesian analyses; and* - *Assessment of model fit.* | 4 |
| Assessment of  Inconsistency | S2 | Describe the statistical methods used to evaluate the agreement of direct and indirect evidence in the treatment network(s) studied. Describe efforts taken to address its presence when found. | 3 |
| Risk of bias across studies | 15 | Specify any assessment of risk of bias that may affect the cumulative evidence (e.g., publication bias, selective reporting within studies). |  |
| Additional analyses | 16 | Describe methods of additional analyses, if done, indicating which were pre- specified. This may include, but not be limited to the following:   - Sensitivity or subgroup analyses; - Meta-regression analyses; - *Alternative formulations of the treatment network; and* - *Use of alternative prior distributions for Bayesian analyses (if applicable).* | 4 |
| **RESULTS** | | |  |
| Study selection | 17 | Give numbers of studies screened, assessed for eligibility, and included in the review, with reasons for exclusions at each stage, ideally with a flow diagram. | 4, 10-12 |
| Presentation of network structure | S3 | Provide a network graph of the included studies to enable visualization of the geometry of the treatment network | 4, figure 2 |
| Summary of network geometry | S4 | Provide a brief overview of characteristics of the treatment network. This may include commentary on the abundance of trials and randomized patients for the different interventions and pairwise comparisons in the network, gaps of evidence in the treatment network, and potential biases reflected by the network structure. | 4 |
| Study characteristics | 18 | For each study, present characteristics for which data were extracted (e.g., study size, PICOS, follow-up period) and provide the citations. | 10-12 |
| Risk of bias within studies | 19 | Present data on risk of bias of each study and, if available, any outcome level assessment (see item 12). | Supplementary material page 22 |
| Results of individual studies | 20 | For all outcomes considered (benefits or harms), present, for each study:1) simple summary data for each intervention group, and 2) effect estimates and confidence/credible intervals. *Modified approaches may be needed to deal with information from larger networks.* | 10-12 |
| Synthesis of results | 21 | Present results of each meta-analysis done, including confidence/credible intervals. *In larger networks, authors may focus on comparisons versus a particular comparator (e.g. placebo or standard care), with full findings presented in an appendix. League tables and forest plots may be considered to summarize pairwise comparisons.* If additional summary measures were explored (such as treatment rankings), these should also be presented. | 4-5, figure 3 |
| Exploration for inconsistency | S5 | Describe results from investigations of inconsistency. This may include such information as measures of model fit to compare consistency and inconsistency models, *P* values from statistical tests, or summary of inconsistency estimates from different parts of the treatment network. | 4, supplementary material page 15-21 |
| Risk of bias across studies | 22 | Present results of any assessment of risk of bias across studies (see Item 15). | Supplementary material page 22 |
| Additional analysis | 23 | Give results of additional analyses, if done (e.g., sensitivity or subgroup analyses, meta-regression, *alternative network geometries studied, alternative choice of prior distributions for Bayesian analyses,* and so forth [see Item 16]). | 5 |
| **DISCUSSION** | | | |
| Summary of evidence | 24 | Summarize the main findings including the strength of evidence for each main outcome; consider their relevance to key groups (e.g., healthcare providers, users, and policy makers). | 5 |
| Limitations | 25 | Discuss limitations at study and outcome level (e.g., risk of bias), and at review-level (e.g., incomplete retrieval of identified research, reporting bias). *Comment on the validity of the assumptions, such as transitivity and consistency. Comment on any concerns regarding network geometry (e.g., avoidance of certain comparisons).* | 6 |
| Conclusions | 26 | Provide a general interpretation of the results in the context of other evidence, and implications for future research. | 6 |
| **FUNDING** | | | |
| Funding | 27 | Describe sources of funding for the systematic review and other support (e.g., supply of data); role of funders for the systematic review. | 7 |

PRISMA = Preferred Reporting Items for Systematic Reviews and Meta-Analysis; PICOS = population, intervention, comparators, outcomes, study design.

*Text in italics indicates wording specific to reporting of network meta-analyses that has been added to guidance from the PRISMA statement.

**Supplementary Table 2 Literature search strategies**

| database | Search strategies | Number of literature |
| --- | --- | --- |
| pubmed | #1= high-grade glioma[Title/Abstract] | 3679 |
|  | #2= malignant glioma[Title/Abstract] | 5488 |
|  | #3= glioblastoma[Title/Abstract] | 42986 |
|  | #4="Glioblastoma"[Mesh] | 30444 |
|  | #5= anaplastic astrocytoma[Title/Abstract] | 2104 |
|  | #6= Anaplastic oligodendroglioma[Title/Abstract] | 620 |
|  | #7= anaplastic oligoastrocytoma[Title/Abstract] | 180 |
|  | #8=#1 OR #2 OR #3 OR #4 OR #5 OR #6 OR #7 | 55586 |
|  | #9= recurren*[Title/Abstract] | 632834 |
|  | #10= relapse[Title/Abstract] | 139454 |
|  | #11= #9 OR #10 | 749922 |
|  | #12= #8 AND #11 | 7807 |
|  | #13= "Temozolomide"[Mesh] | 5580 |
|  | #14= temozolomide[Title/Abstract] | 8989 |
|  | #15= lomustine[Title/Abstract] | 1013 |
|  | #16= "Lomustine"[Mesh] | 2340 |
|  | #17= carmustine[Title/Abstract] | 1664 |
|  | #18= "Carmustine"[Mesh] | 4077 |
|  | #19= procarbazine[Title/Abstract] | 2266 |
|  | #20= "Procarbazine"[Mesh] | 3293 |
|  | #21= vincristine[Title/Abstract] | 20114 |
|  | #22= "Vincristine"[Mesh] | 24284 |
|  | #23= PCV[Title/Abstract] | 7489 |
|  | #24= bevacizumab[Title/Abstract] | 18853 |
|  | #25= "Bevacizumab"[Mesh] | 13472 |
|  | #26= regorafenib[Title/Abstract] | 1615 |
|  | #27= #13 OR #14 OR #15 OR #16 OR #17 OR #18 OR #19 OR #20 OR #21 OR #22 OR #23 OR #24 OR #25 OR #26 | 77764 |
|  | #28= randomized controlled trial[Publication Type] | 574843 |
|  | #29= controlled clinical trial[Publication Type] | 664902 |
|  | #30= (((((randomized[Title/Abstract]) OR (controlled[Title/Abstract])) OR (trial[Title/Abstract])) OR (random[Title/Abstract])) OR (placebo[Title/Abstract])) OR (groups[Title/Abstract]) | 3935728 |
|  | #31= #28 OR #29 OR #30 | 4077132 |
|  | #32= #12 AND #27 AND #31 | 754 |
| Embase | **#1** high-grade glioma:ti,ab,kw | 6498 |
|  | **#2** malignant glioma:ti,ab,kw | 7709 |
|  | **#3** glioblastoma:ti,ab,kw | 65540 |
|  | **#4** "Glioblastoma":ti,ab,kw | 30444 |
|  | **#5** 'anaplastic astrocytoma':ti,ab,kw OR 'anaplastic oligodendroglioma':ti,ab,kw OR 'anaplastic oligoastrocytoma':ti,ab,kw | 4198 |
|  | **#6** #1 OR #2 OR #3 OR #4 OR #5 OR #6 OR #7 | 55586 |
|  | **#7** recurren*:ti,ab,kw OR relapse:ti,ab,kw | 234026 |
|  | **#8**= #6 AND #7 | 15509 |
|  | **#9** 'temozolomide'/exp OR 'lomustine'/exp OR 'carmustine'/exp OR 'procarbazine'/exp OR 'vincristine'/exp OR 'bevacizumab'/exp OR 'regorafenib'/exp | 217441 |
|  | **#10** temozolomide:ti,ab,kw OR lomustine:ti,ab,kw OR carmustine:ti,ab,kw OR procarbazine:ti,ab,kw OR vincristine:ti,ab,kw OR pcv:ti,ab,kw OR bevacizumab:ti,ab,kw OR regorafenib:ti,ab,kw | 93505 |
|  | **#11** #9 OR #10 | 234026 |
|  | **#12** randomized:ti,ab,kw OR controlled:ti,ab,kw OR trial:ti,ab,kw OR random:ti,ab,kw OR placebo:ti,ab,kw OR groups:ti,ab,kw | 5382684 |
|  | **#13** 'controlled clinical trial' | 465044 |
|  | **#14** 'randomized controlled trial' | 969443 |
|  | **#15** #12 OR #13 OR #14 | 5683023 |
|  | **#16**= #8 AND #11 AND #15 | 2360 |
| Cochrane Central Register of Controlled Trials | #1= (high-grade glioma) :ti,ab,kw | 3679 |
|  | #2= (malignant glioma):ti,ab,kw | 5488 |
|  | #3= (glioblastoma):ti,ab,kw | 42986 |
|  | #4= MeSh descriptor: [Glioblastoma] explode all trees | 30444 |
|  | #5= anaplastic astrocytoma:ti,ab,kw | 2104 |
|  | #6= (Anaplastic oligodendroglioma):ti,ab,kw | 620 |
|  | #7=( anaplastic oligoastrocytoma):ti,ab,kw | 180 |
|  | #8=#1 OR #2 OR #3 OR #4 OR #5 OR #6 OR #7 | 55586 |
|  | #9= (recurren*):ti,ab,kw | 632834 |
|  | #10= (relapse):ti,ab,kw | 139454 |
|  | #11= #9 OR #10 | 749922 |
|  | #12= #8 AND #11 | 7807 |
|  | #13= MeSh descriptor: [Temozolomide] explode all trees | 5580 |
|  | #14= (temozolomide):ti,ab,kw | 8989 |
|  | #15= (lomustine):ti,ab,kw | 1013 |
|  | #16= MeSh descriptor: [Lomustine] explode all trees | 2340 |
|  | #17= (carmustine):ti,ab,kw | 1664 |
|  | #18= MeSh descriptor: [Carmustine] explode all trees | 4077 |
|  | #19= (procarbazine):ti,ab,kw | 2266 |
|  | #20= MeSh descriptor: [Procarbazine] explode all trees | 3293 |
|  | #21= (vincristine):ti,ab,kw | 20114 |
|  | #22= MeSh descriptor: [Vincristine] explode all trees | 24284 |
|  | #23= PCV:ti,ab,kw | 7489 |
|  | #24= (bevacizumab):ti,ab,kw | 18853 |
|  | #25= MeSh descriptor: [Bevacizumab] explode all trees | 13472 |
|  | #26= (regorafenib):ti,ab,kw | 1615 |
|  | #21= #7 OR #8 OR #9 OR #10 OR #11 OR #12 OR #13 OR #14 OR #15 OR #16 OR #17 OR #18 OR #19 OR #20 | 14898 |
|  | #28= randomized controlled trial[Publication Type] | 574843 |
|  | #29= controlled clinical trial[Publication Type] | 664902 |
|  | #30= (randomized):ti,ab,kw OR (controlled):ti,ab,kw OR (tria)l:ti,ab,kw OR (random):ti,ab,kw OR (placebo):ti,ab,kw OR (groups):ti,ab,kw | 3935728 |
|  | #31= #28 OR #29 OR #30 | 4077132 |
|  | #32= #12 AND #27 AND #31 | 663 |

**Supplementary table 3 Bayesian ranking results of network meta-analysis**

| Treatments | Ranking of probability | | | | | | | | | | |
| --- | --- | --- | --- | --- | --- | --- | --- | --- | --- | --- | --- |
|  | 1 | 2 | 3 | 4 | 5 | 6 | 7 | 8 | 9 | 10 | 11 |
| Overall survival | | | | | | | | | | | |
| Bevacizumab | 0.00% | 0.10% | 0.70% | 2.94% | 9.38% | 20.68% | 29.22% | 24.27% | 10.49% | 2.09% | 0.14% |
| Bevacizumab plus carboplatin | 0.02% | 0.58% | 1.31% | 2.58% | 4.07% | 5.11% | 5.71% | 8.76% | 18.62% | 39.21% | 14.03% |
| Bevacizumab plus dasatinib | 1.06% | 12.79% | 11.66% | 16.43% | 16.56% | 10.51% | 6.56% | 7.30% | 8.70% | 6.72% | 1.70% |
| Bevacizumab plus irinotecan | 0.01% | 0.28% | 1.56% | 5.29% | 13.45% | 23.41% | 25.75% | 18.80% | 9.02% | 2.25% | 0.19% |
| Bevacizumab plus lomustine (90mg/m^2^) | 0.19% | 33.78% | 28.36% | 16.09% | 7.83% | 3.80% | 3.05% | 3.13% | 2.49% | 1.17% | 0.12% |
| Bevacizumab plus lomustine (110mg/m^2^) | 0.48% | 2.83% | 1.60% | 2.81% | 2.89% | 2.27% | 1.66% | 1.84% | 3.54% | 7.87% | 72.22% |
| Bevacizumab plus temozolomide | 2.23% | 17.43% | 10.81% | 14.73% | 12.96% | 8.21% | 5.27% | 6.55% | 9.08% | 9.61% | 3.12% |
| Bevacizumab plus vorinostat | 1.96% | 14.09% | 9.19% | 12.26% | 11.63% | 7.89% | 5.28% | 6.86% | 10.87% | 14.40% | 5.58% |
| Lomustine | 0.01% | 12.85% | 31.90% | 21.80% | 12.26% | 5.91% | 3.89% | 4.20% | 4.25% | 2.65% | 0.28% |
| Nivolumab | 0.02% | 0.67% | 2.01% | 4.84% | 8.91% | 12.15% | 13.57% | 18.26% | 22.94% | 14.02% | 2.63% |
| Regorafenib | 94.04% | 4.60% | 0.92% | 0.24% | 0.07% | 0.05% | 0.04% | 0.03% | 0.01% | 0.01% | 0.00% |
| Progression-free survival | | | | | | | | | | | |
| Bevacizumab | 0.06% | 0.52% | 2.13% | 5.67% | 11.51% | 18.10% | 22.67% | 21.70% | 13.61% | 3.79% | 0.25% |
| Bevacizumab plus carboplatin | 5.44% | 7.41% | 8.66% | 9.69% | 10.51% | 10.73% | 11.06% | 11.18% | 10.92% | 9.89% | 4.51% |
| Bevacizumab plus dasatinib | 10.16% | 11.63% | 12.14% | 12.47% | 11.75% | 10.67% | 9.01% | 7.72% | 6.42% | 5.43% | 2.59% |
| Bevacizumab plus irinotecan | 3.83% | 10.52% | 11.62% | 12.05% | 11.72% | 11.36% | 10.58% | 9.68% | 9.16% | 7.05% | 2.42% |
| Bevacizumab plus lomustine (90mg/m^2^) | 11.73% | 17.88% | 17.82% | 15.14% | 12.27% | 9.27% | 7.01% | 5.11% | 2.76% | 0.86% | 0.15% |
| Bevacizumab plus lomustine (110mg/m^2^) | 16.88% | 11.45% | 10.50% | 9.65% | 8.77% | 7.79% | 7.55% | 7.63% | 8.33% | 7.11% | 4.33% |
| Bevacizumab plus temozolomide | 18.96% | 12.63% | 10.40% | 9.35% | 8.27% | 7.49% | 6.83% | 6.62% | 6.92% | 7.18% | 5.35% |
| Bevacizumab plus vorinostat | 24.09% | 17.93% | 14.34% | 11.62% | 9.10% | 6.77% | 5.05% | 4.00% | 3.23% | 2.57% | 1.30% |
| Lomustine | 0.03% | 0.33% | 1.31% | 2.92% | 4.37% | 5.85% | 7.68% | 11.18% | 18.32% | 34.21% | 13.79% |
| Nivolumab | 0.44% | 0.79% | 1.07% | 1.41% | 1.81% | 2.44% | 3.29% | 5.07% | 8.46% | 14.87% | 60.34% |
| Regorafenib | 8.38% | 8.91% | 10.00% | 10.03% | 9.92% | 9.52% | 9.27% | 10.10% | 11.88% | 7.03% | 4.96% |
| Objective response rate | | | | | | | | | | | |
| Bevacizumab | 0.06% | 1.35% | 11.24% | 35.50% | 36.13% | 13.21% | 2.38% | 0.14% | 0.00% | 0.00% |  |
| Bevacizumab plus carboplatin | 35.98% | 35.38% | 12.89% | 7.43% | 4.07% | 2.64% | 1.24% | 0.33% | 0.04% | 0.01% |  |
| Bevacizumab plus dasatinib | 0.15% | 0.81% | 2.35% | 4.15% | 9.04% | 20.14% | 34.86% | 21.03% | 6.03% | 1.44% |  |
| Bevacizumab plus irinotecan | 7.20% | 28.89% | 43.41% | 14.27% | 4.63% | 1.37% | 0.22% | 0.02% | 0.00% | 0.00% |  |
| Bevacizumab plus lomustine (90mg/m2) | 0.16% | 1.74% | 6.17% | 10.80% | 20.40% | 32.56% | 22.30% | 5.43% | 0.44% | 0.00% |  |
| Bevacizumab plus lomustine (110mg/m2) | 53.56% | 24.30% | 9.08% | 5.62% | 3.63% | 2.17% | 1.15% | 0.39% | 0.08% | 0.01% |  |
| Bevacizumab plus temozolomide | 1.50% | 5.27% | 12.12% | 19.54% | 18.61% | 20.13% | 15.17% | 6.01% | 1.38% | 0.27% |  |
| Lomustine | 0.00% | 0.00% | 0.00% | 0.00% | 0.00% | 0.05% | 0.63% | 5.34% | 34.96% | 59.02% |  |
| Nivolumab | 0.00% | 0.00% | 0.00% | 0.03% | 0.31% | 2.21% | 11.23% | 43.82% | 32.11% | 10.28% |  |
| Regorafenib | 1.40% | 2.26% | 2.74% | 2.66% | 3.17% | 5.52% | 10.82% | 17.49% | 24.94% | 28.99% |  |
| Grade ≥3 adverse events | | | | | | | | | | | |
| Bevacizumab | 1.05% | 16.44% | 42.88% | 30.45% | 8.30% | 0.85% | 0.01% | 0.00% |  | | |
| Bevacizumab plus carboplatin | 1.64% | 11.38% | 14.66% | 20.64% | 25.36% | 18.11% | 7.25% | 0.96% |  |  |  |
| Bevacizumab plus dasatinib | 0.97% | 6.53% | 8.55% | 12.20% | 19.64% | 26.15% | 20.43% | 5.53% |  |  |  |
| Bevacizumab plus irinotecan | 0.04% | 0.36% | 1.07% | 3.99% | 11.60% | 28.34% | 53.84% | 0.77% |  |  |  |
| Bevacizumab plus temozolomide | 0.00% | 0.02% | 0.04% | 0.14% | 0.43% | 1.45% | 7.06% | 90.85% |  |  |  |
| Bevacizumab plus vorinostat | 10.61% | 46.56% | 16.13% | 12.04% | 8.66% | 4.46% | 1.37% | 0.18% |  |  |  |
| Fotemustine | 84.00% | 7.29% | 2.66% | 2.12% | 1.82% | 1.22% | 0.71% | 0.18% |  |  |  |
| Nivolumab | 1.68% | 11.43% | 14.01% | 18.42% | 24.19% | 19.42% | 9.32% | 1.53% |  |  |  |

**Supplementary Table 4 Surface under the cumulative ranking curve results of network meta-analysis**

| Treatments | Ranking of cumulative probability | | | | | | | | | | |
| --- | --- | --- | --- | --- | --- | --- | --- | --- | --- | --- | --- |
|  | 1 | 2 | 3 | 4 | 5 | 6 | 7 | 8 | 9 | 10 | 11 |
| Overall survival | | | | | | | | | | | |
| Bevacizumab | 0.00% | 0.10% | 0.80% | 3.74% | 13.11% | 33.79% | 63.02% | 87.29% | 97.77% | 99.86% | 100.00% |
| Bevacizumab plus carboplatin | 0.02% | 0.60% | 1.91% | 4.49% | 8.56% | 13.67% | 19.38% | 28.14% | 46.76% | 85.97% | 100.00% |
| Bevacizumab plus dasatinib | 1.06% | 13.85% | 25.51% | 41.94% | 58.50% | 69.01% | 75.58% | 82.88% | 91.58% | 98.30% | 100.00% |
| Bevacizumab plus irinotecan | 0.01% | 0.28% | 1.84% | 7.13% | 20.58% | 43.99% | 69.75% | 88.55% | 97.56% | 99.81% | 100.00% |
| Bevacizumab plus lomustine (90mg/m^2^) | 0.19% | 33.97% | 62.33% | 78.42% | 86.25% | 90.05% | 93.09% | 96.22% | 98.71% | 99.88% | 100.00% |
| Bevacizumab plus lomustine (110mg/m^2^) | 0.48% | 3.30% | 4.90% | 7.71% | 10.60% | 12.88% | 14.53% | 16.37% | 19.91% | 27.78% | 100.00% |
| Bevacizumab plus temozolomide | 2.23% | 19.66% | 30.47% | 45.20% | 58.16% | 66.37% | 71.64% | 78.19% | 87.26% | 96.88% | 100.00% |
| Bevacizumab plus vorinostat | 1.96% | 16.04% | 25.23% | 37.49% | 49.12% | 57.01% | 62.29% | 69.15% | 80.02% | 94.43% | 100.00% |
| Lomustine | 0.01% | 12.86% | 44.76% | 66.55% | 78.82% | 84.73% | 88.62% | 92.82% | 97.07% | 99.72% | 100.00% |
| Nivolumab | 0.02% | 0.69% | 2.70% | 7.53% | 16.44% | 28.59% | 42.16% | 60.41% | 83.35% | 97.37% | 100.00% |
| Regorafenib | 94.04% | 98.64% | 99.56% | 99.80% | 99.87% | 99.92% | 99.96% | 99.99% | 99.99% | 100.00% | 100.00% |
| Progression-free survival | | | | | | | | | | | |
| Bevacizumab | 0.06% | 0.58% | 2.70% | 8.38% | 19.89% | 37.98% | 60.65% | 82.35% | 95.96% | 99.75% | 100.00% |
| Bevacizumab plus carboplatin | 5.44% | 12.85% | 21.51% | 31.20% | 41.71% | 52.44% | 63.50% | 74.68% | 85.60% | 95.49% | 100.00% |
| Bevacizumab plus dasatinib | 10.16% | 21.79% | 33.93% | 46.41% | 58.16% | 68.83% | 77.84% | 85.56% | 91.98% | 97.41% | 100.00% |
| Bevacizumab plus irinotecan | 3.83% | 14.36% | 25.98% | 38.02% | 49.74% | 61.10% | 71.69% | 81.37% | 90.53% | 97.58% | 100.00% |
| Bevacizumab plus lomustine (90mg/m^2^) | 11.73% | 29.60% | 47.42% | 62.56% | 74.83% | 84.10% | 91.11% | 96.22% | 98.99% | 99.85% | 100.00% |
| Bevacizumab plus lomustine (110mg/m^2^) | 16.88% | 28.33% | 38.83% | 48.49% | 57.26% | 65.05% | 72.60% | 80.23% | 88.56% | 95.67% | 100.00% |
| Bevacizumab plus temozolomide | 18.96% | 31.60% | 42.00% | 51.34% | 59.61% | 67.10% | 73.94% | 80.55% | 87.47% | 94.65% | 100.00% |
| Bevacizumab plus vorinostat | 24.09% | 42.02% | 56.37% | 67.99% | 77.09% | 83.86% | 88.91% | 92.91% | 96.13% | 98.70% | 100.00% |
| Lomustine | 0.03% | 0.36% | 1.67% | 4.59% | 8.96% | 14.81% | 22.49% | 33.67% | 51.99% | 86.21% | 100.00% |
| Nivolumab | 0.44% | 1.23% | 2.30% | 3.71% | 5.52% | 7.96% | 11.25% | 16.32% | 24.79% | 39.66% | 100.00% |
| Regorafenib | 8.38% | 17.28% | 27.28% | 37.32% | 47.24% | 56.76% | 66.03% | 76.13% | 88.01% | 95.04% | 100.00% |
| Objective response rate | | | | | | | | | | | |
| Bevacizumab | 0.06% | 1.41% | 12.65% | 48.14% | 84.27% | 97.48% | 99.87% | 100.00% | 100.00% | 100.00% |  |
| Bevacizumab plus carboplatin | 35.98% | 71.36% | 84.25% | 91.68% | 95.75% | 98.39% | 99.63% | 99.95% | 99.99% | 100.00% |  |
| Bevacizumab plus dasatinib | 0.15% | 0.96% | 3.31% | 7.46% | 16.50% | 36.64% | 71.50% | 92.53% | 98.57% | 100.00% |  |
| bevacizumab plus irinotecan | 7.20% | 36.09% | 79.49% | 93.76% | 98.39% | 99.76% | 99.98% | 100.00% | 100.00% | 100.00% |  |
| Bevacizumab plus lomustine (90mg/m^2^) | 0.16% | 1.90% | 8.07% | 18.87% | 39.28% | 71.83% | 94.13% | 99.56% | 100.00% | 100.00% |  |
| Bevacizumab plus lomustine (110mg/m^2^) | 53.56% | 77.86% | 86.94% | 92.57% | 96.20% | 98.37% | 99.52% | 99.91% | 99.99% | 100.00% |  |
| Bevacizumab plus temozolomide | 1.50% | 6.77% | 18.89% | 38.43% | 57.03% | 77.17% | 92.34% | 98.35% | 99.73% | 100.00% |  |
| Lomustine | 0.00% | 0.00% | 0.00% | 0.00% | 0.00% | 0.05% | 0.68% | 6.02% | 40.98% | 100.00% |  |
| Nivolumab | 0.00% | 0.00% | 0.00% | 0.03% | 0.34% | 2.56% | 13.79% | 57.61% | 89.72% | 100.00% |  |
| Regorafenib | 1.40% | 3.66% | 6.40% | 9.06% | 12.23% | 17.75% | 28.57% | 46.07% | 71.01% | 100.00% |  |
| Grade ≥ 3 adverse events | | | | | | | | | | | |
| Bevacizumab | 1.05% | 17.50% | 60.38% | 90.83% | 99.13% | 99.98% | 100.00% | 100.00% |  | | |
| Bevacizumab plus carboplatin | 1.64% | 13.02% | 27.68% | 48.32% | 73.68% | 91.79% | 99.04% | 100.00% |  |  |  |
| Bevacizumab plus dasatinib | 0.97% | 7.50% | 16.04% | 28.25% | 47.89% | 74.04% | 94.47% | 100.00% |  |  |  |
| Bevacizumab plus irinotecan | 0.04% | 0.40% | 1.47% | 5.46% | 17.06% | 45.39% | 99.23% | 100.00% |  |  |  |
| Bevacizumab plus temozolomide | 0.00% | 0.02% | 0.07% | 0.20% | 0.64% | 2.09% | 9.15% | 100.00% |  |  |  |
| Bevacizumab plus vorinostat | 10.61% | 57.17% | 73.30% | 85.34% | 93.99% | 98.45% | 99.82% | 100.00% |  |  |  |
| Fotemustine | 84.00% | 91.29% | 93.94% | 96.07% | 97.88% | 99.10% | 99.82% | 100.00% |  |  |  |
| Nivolumab | 1.68% | 13.11% | 27.12% | 45.54% | 69.73% | 89.15% | 98.47% | 100.00% |  |  |  |


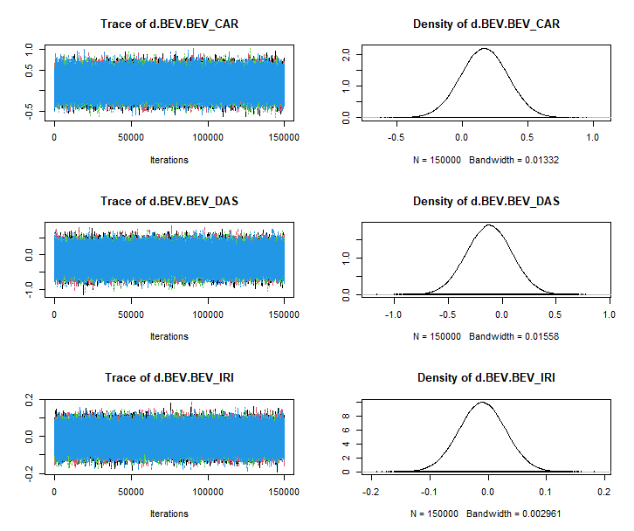

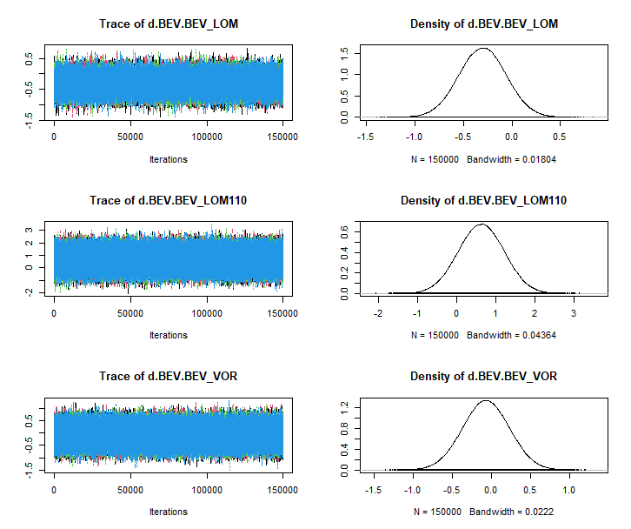

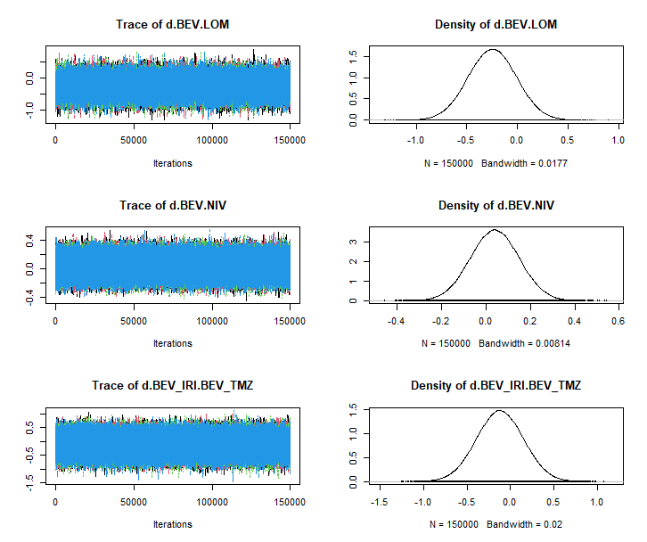


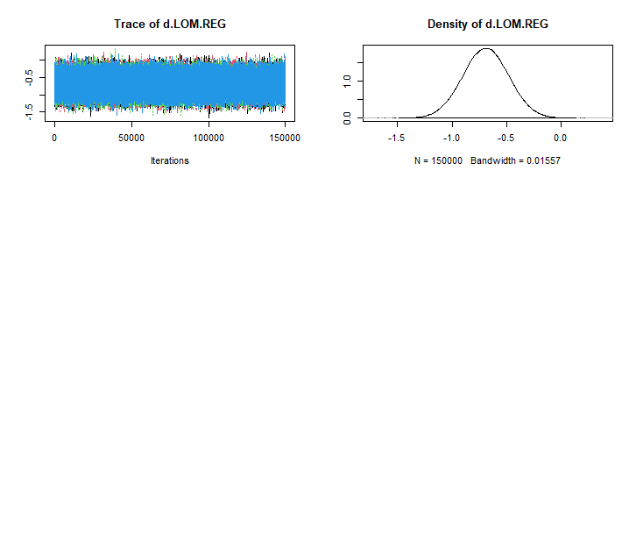


**A Density plots and trace plots for overall survival**

**
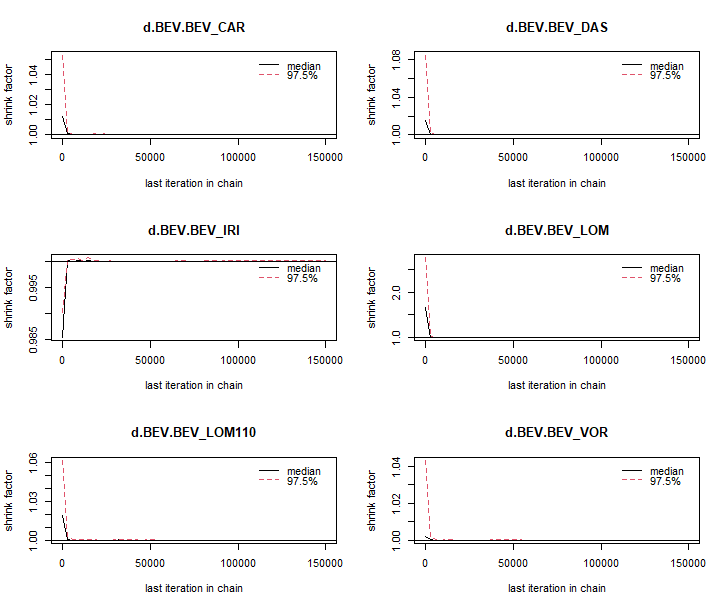

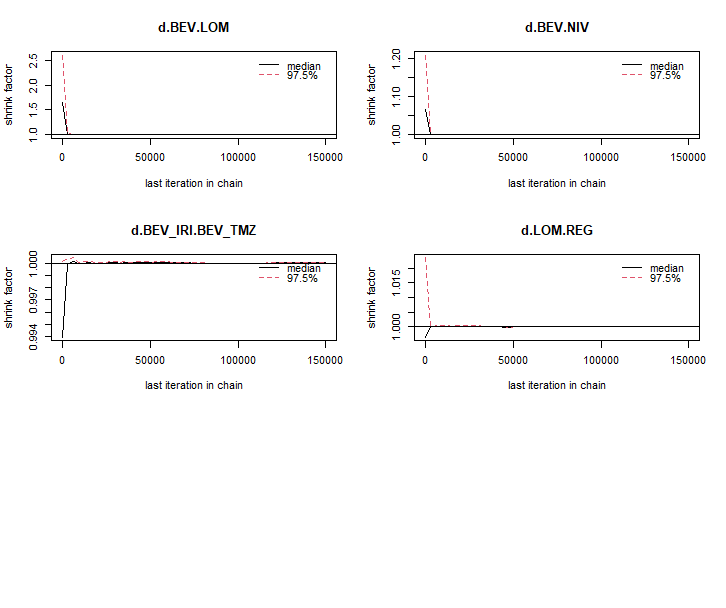
B Brooks-Gelman Rubin diagnosis plots for overall survival**

**
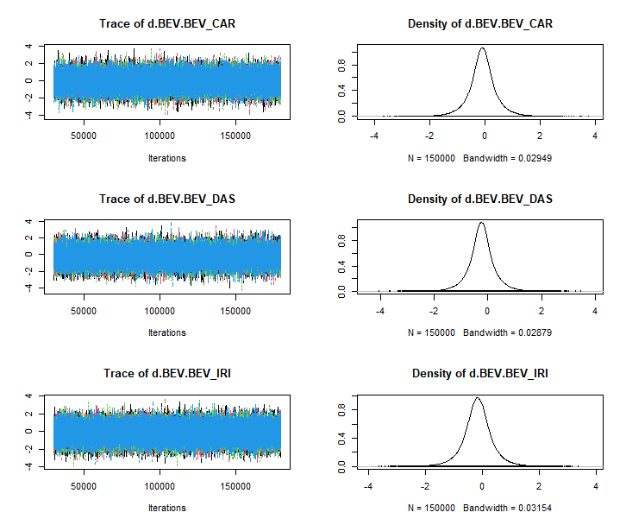

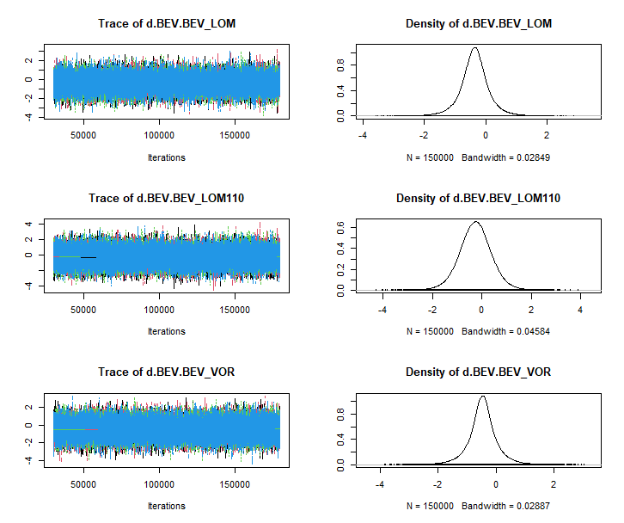

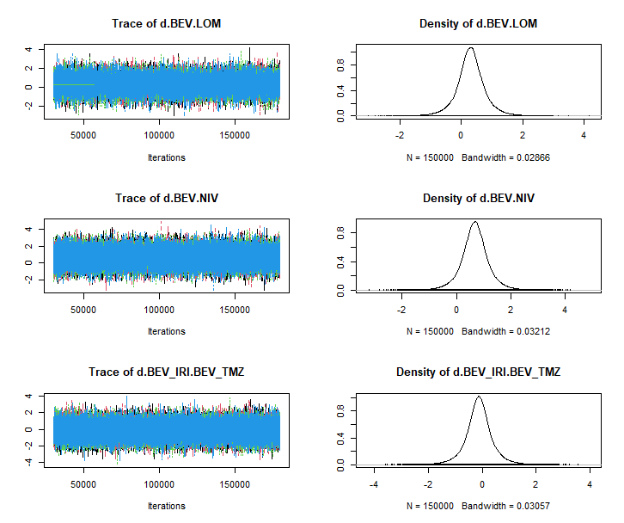
**

**
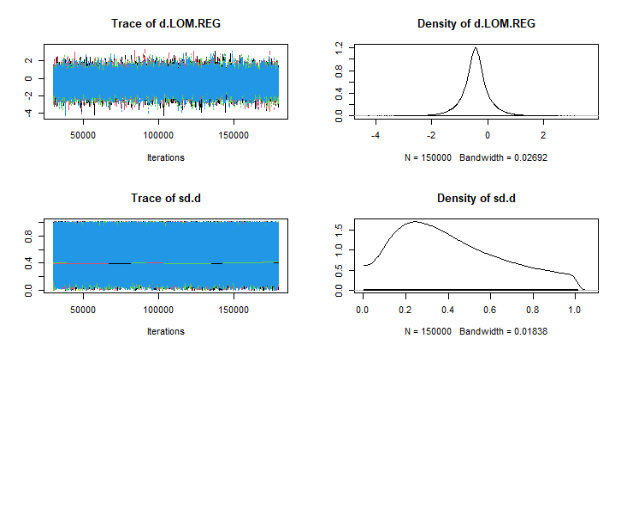
**

**C Density plots and trace plots for progression-free survival**


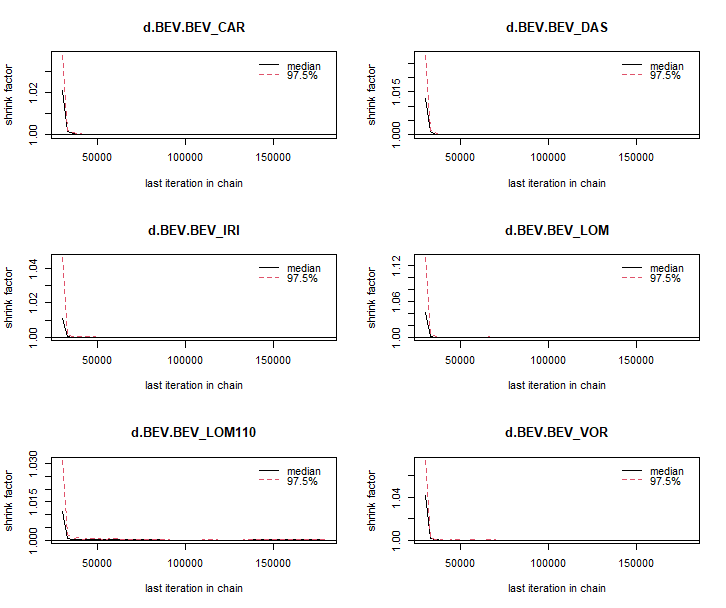

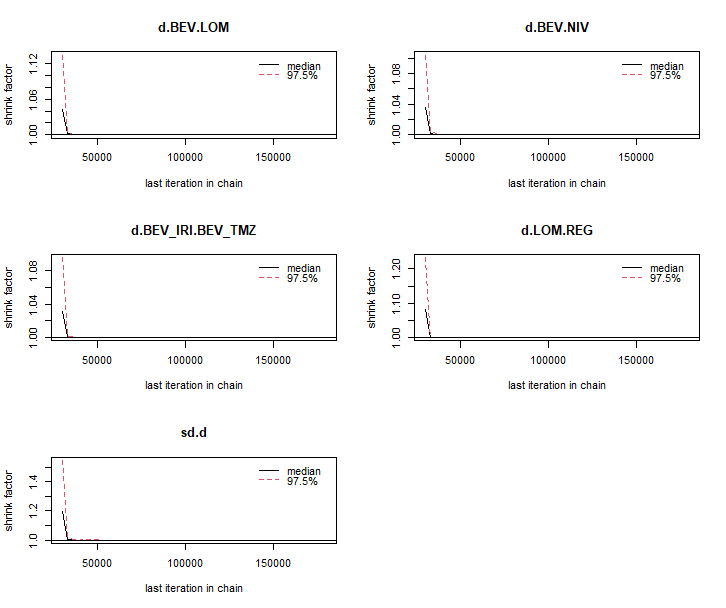


**D Brooks-Gelman Rubin diagnosis plots for progression-free survival**


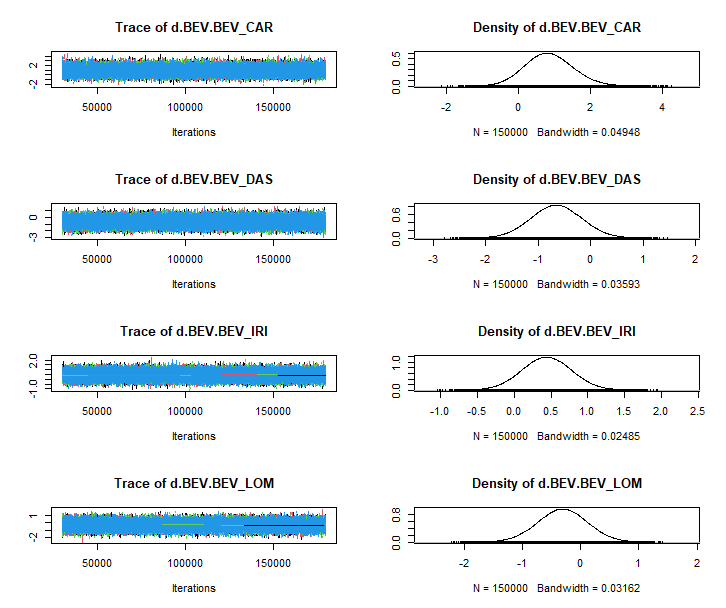

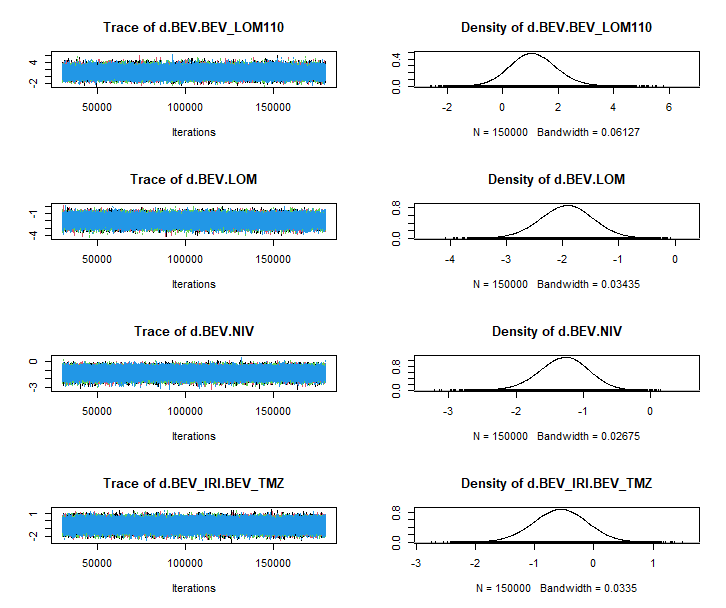

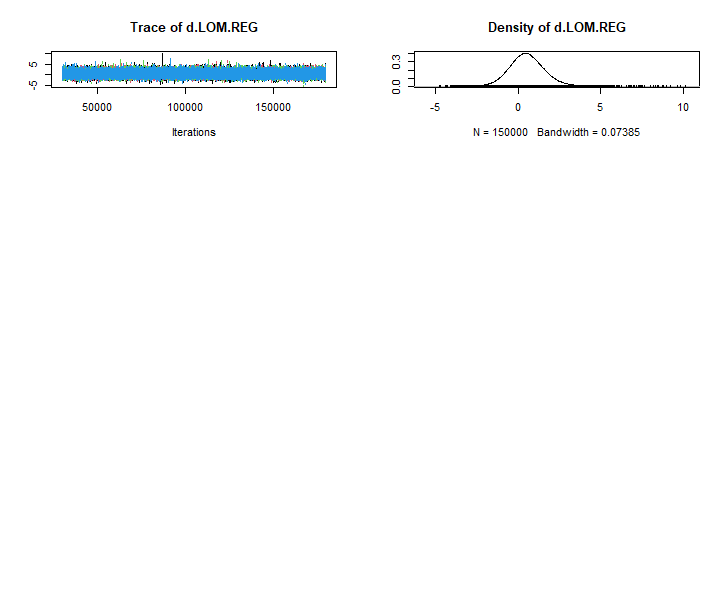


**E Density plots and trace plots for objective response rate**


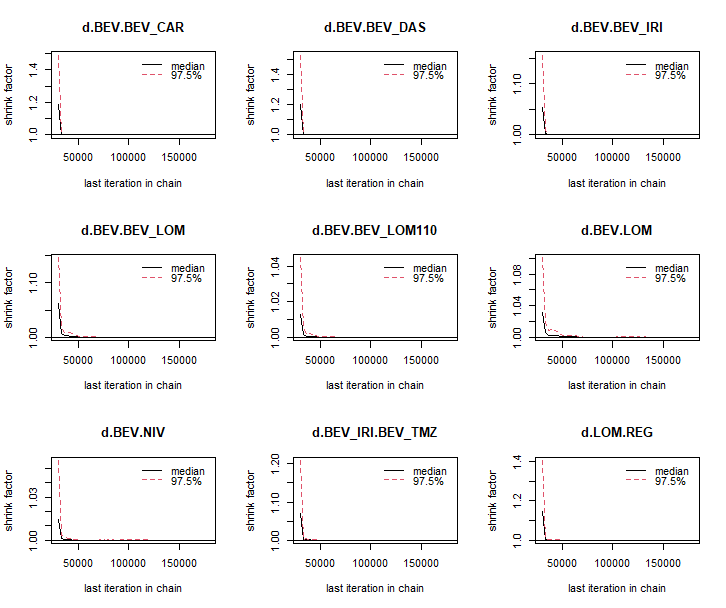


**F Brooks-Gelman Rubin diagnosis plots for objective response rate**


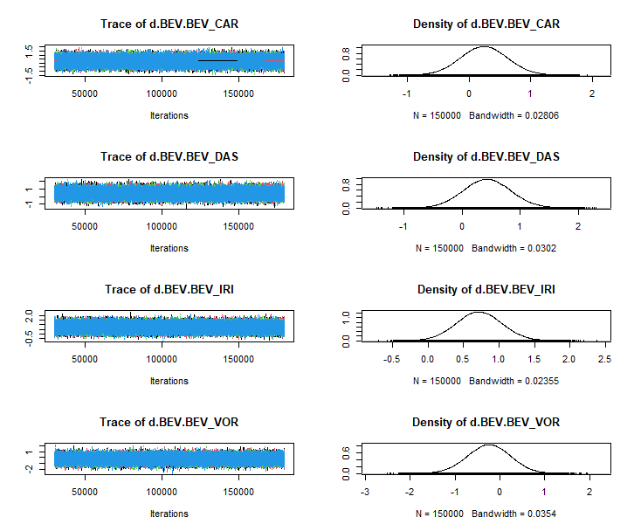

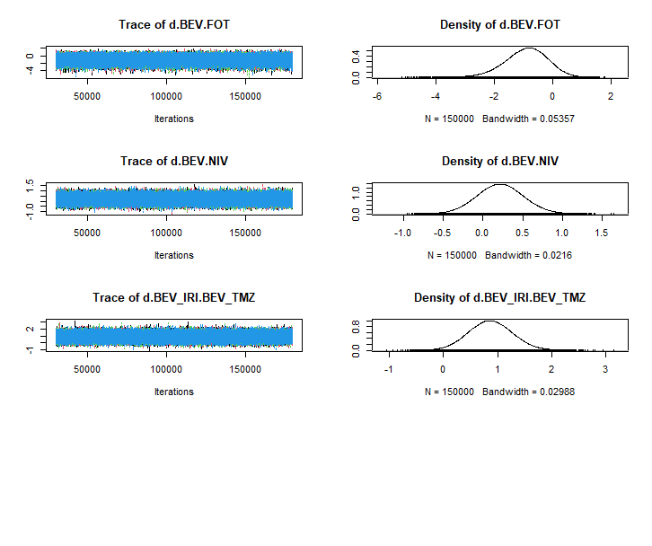


**G Density plots and trace plots for 3 or higher adverse events.**


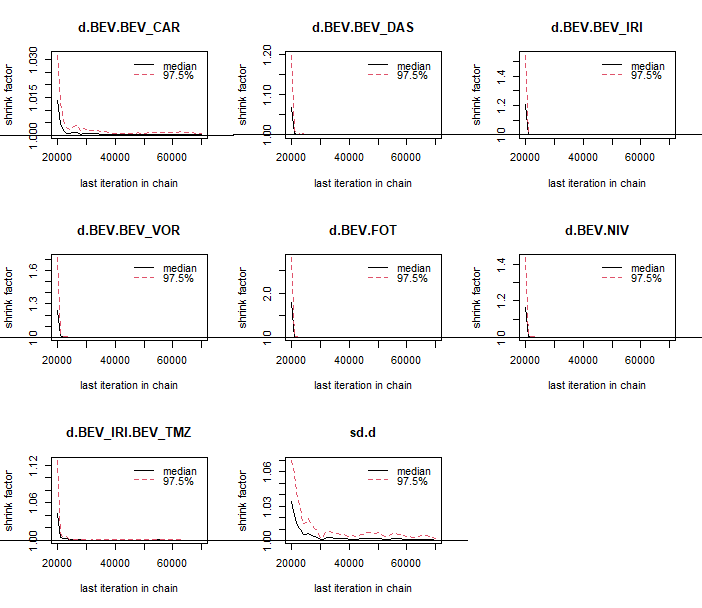


**H Brooks-Gelman Rubin diagnosis plots for objective 3 or higher adverse events**

**Supplementary figure 1 Convergence of the three chains established by trace and the Brooks-Gelman-Rubin diagnostic for overall survival (A and B), progression-free survival (C and D), objective response rate (E and F), and 3 or higher adverse events (G and H).**

A

**B**

**Supplementary Figure 2 Results of risk of bias assessment using the Cochrane risk of bias tool 2.** (A) risk of bias summary. (B) Risk of bias of the literature

**A**

**
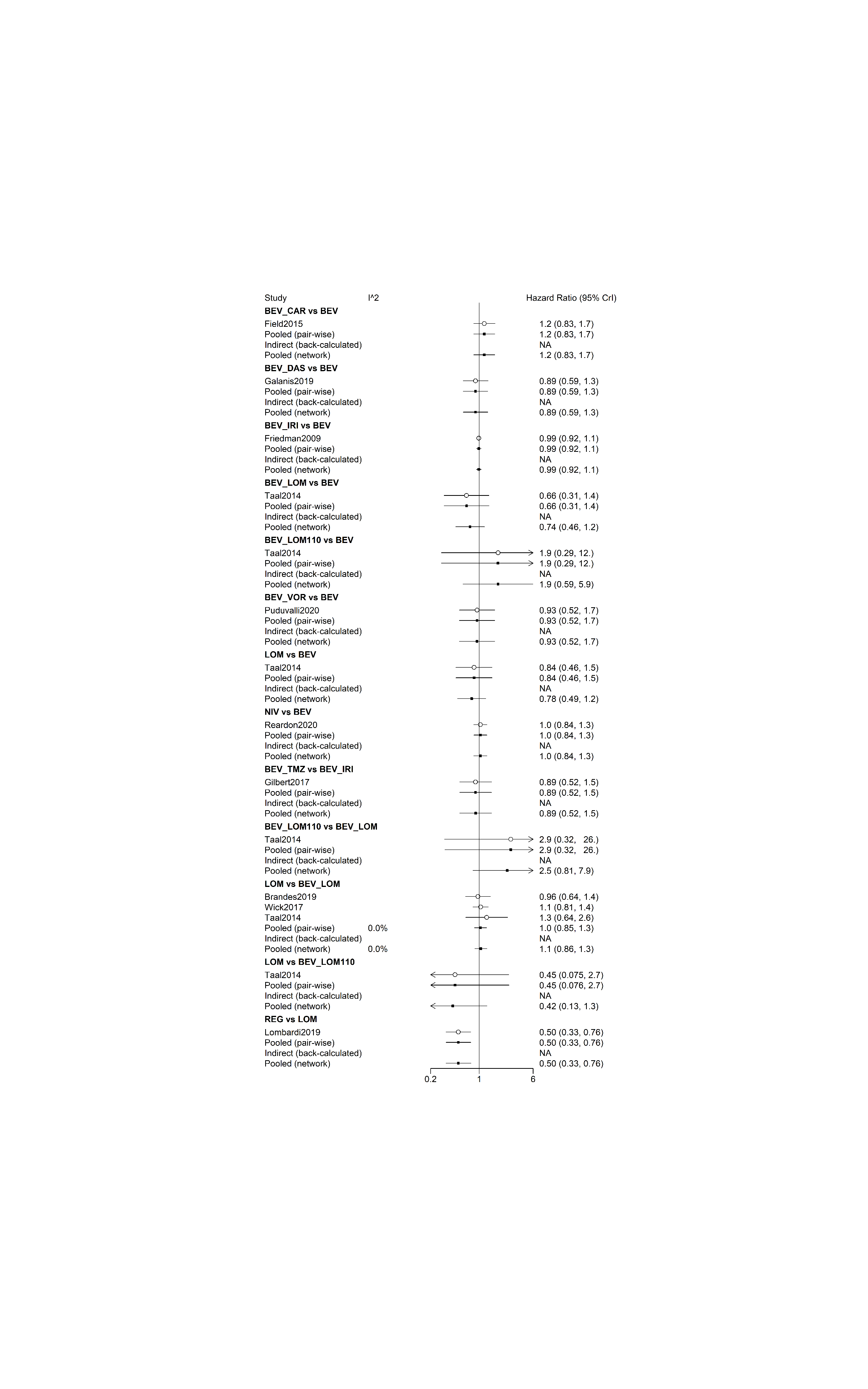
**

**B**


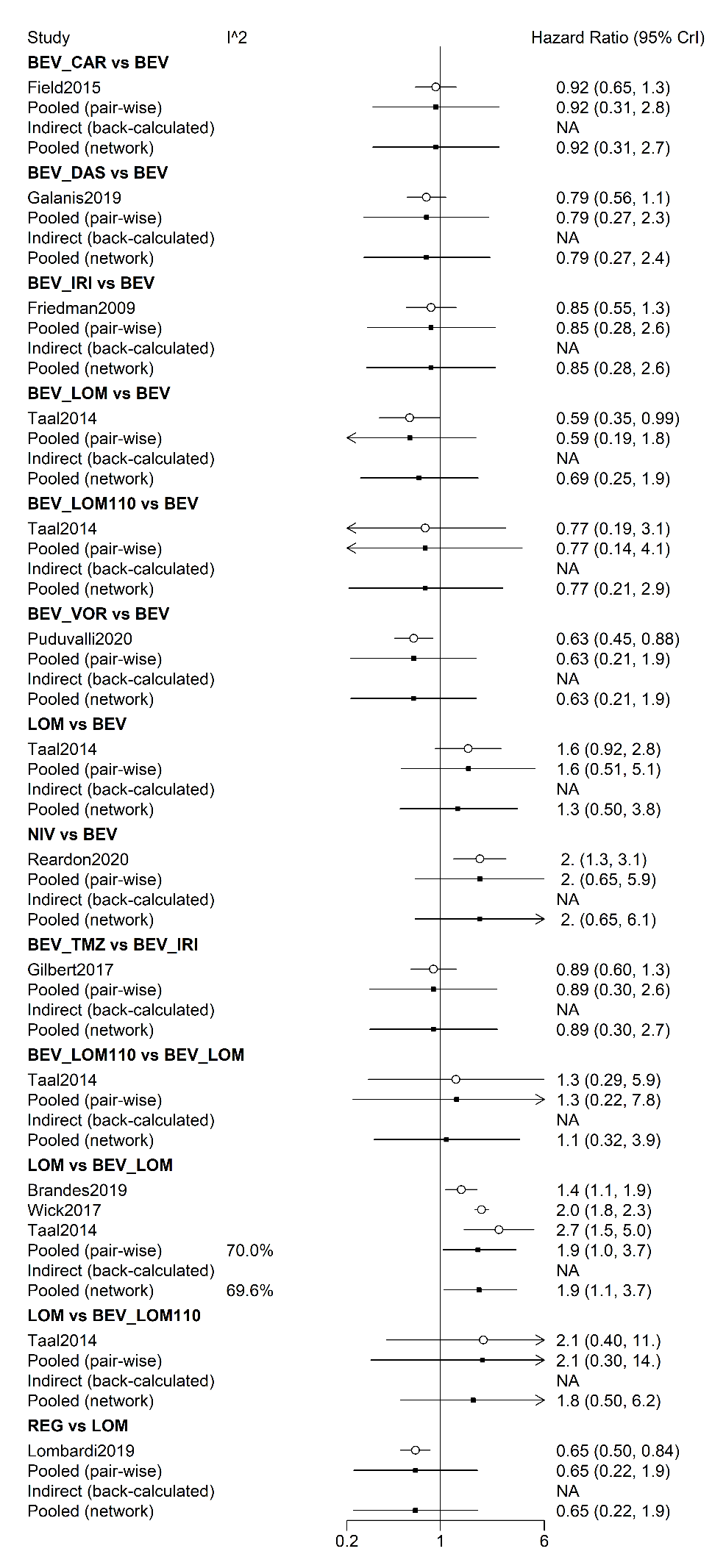


**C**

**
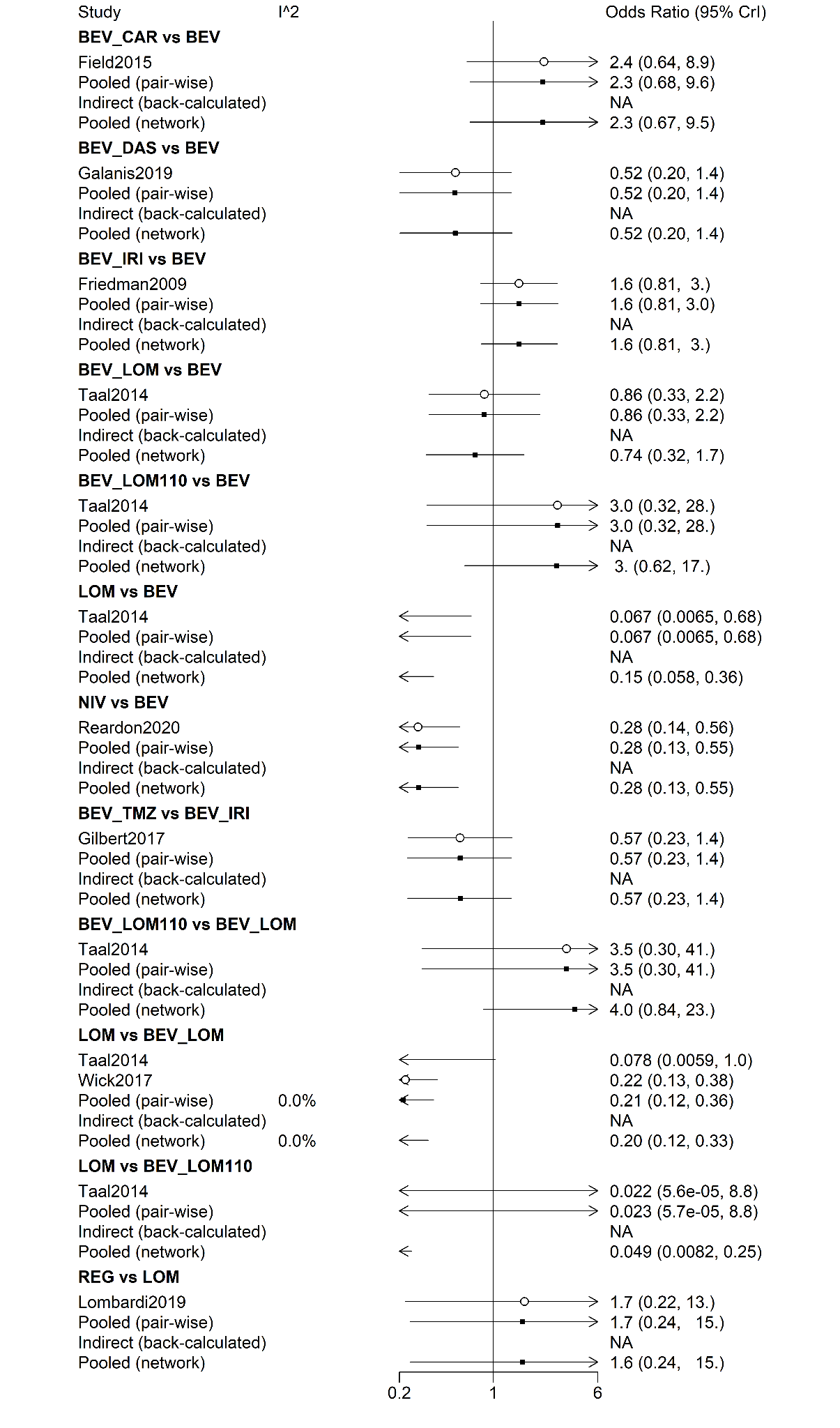
**

**D**

**
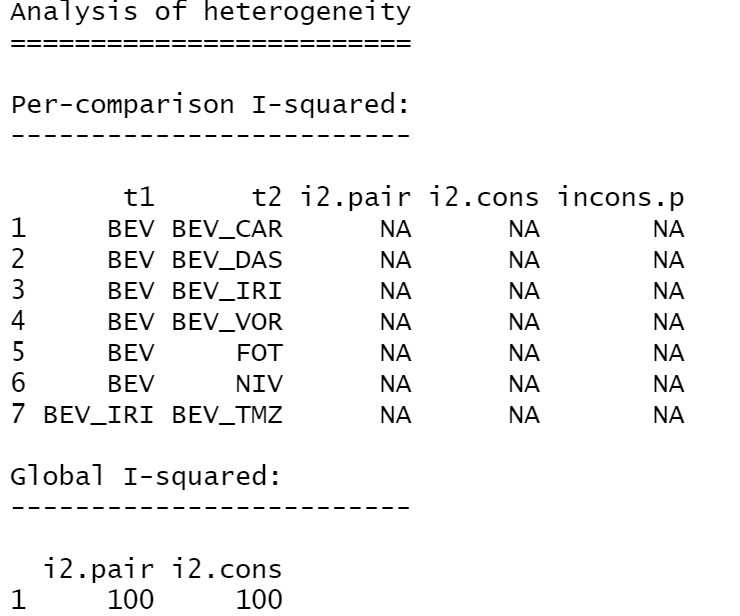
**

**
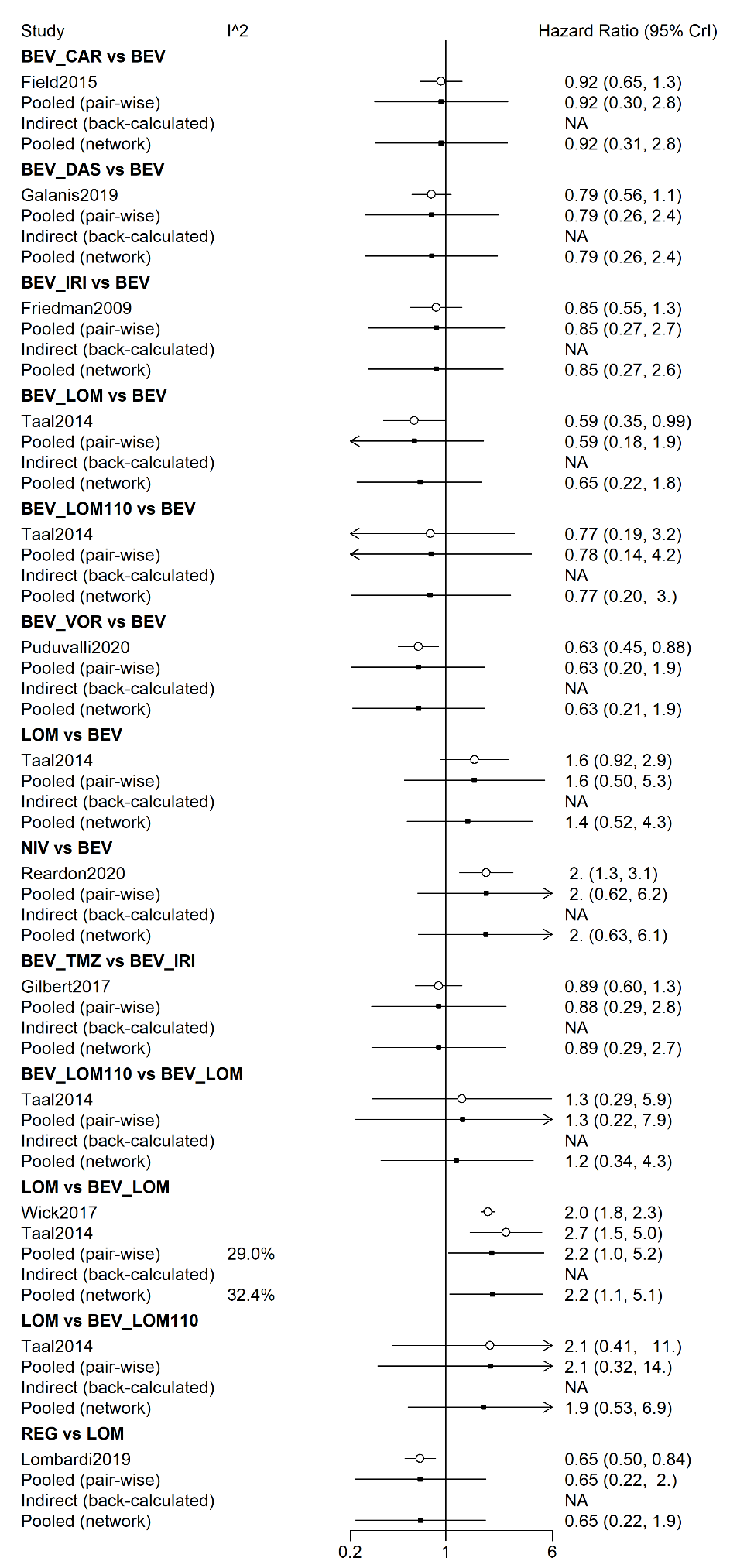
E**

**Supplementary figure 3 Forest plots and I^2^ of head-to-head comparisons** (A) Results of overall survival. (B) Results of progression-free survival. (C) Results of objective response rate. (D) Results of grade 3 or higher adverse events; (E) Results of sensitivity analysis.

**A**
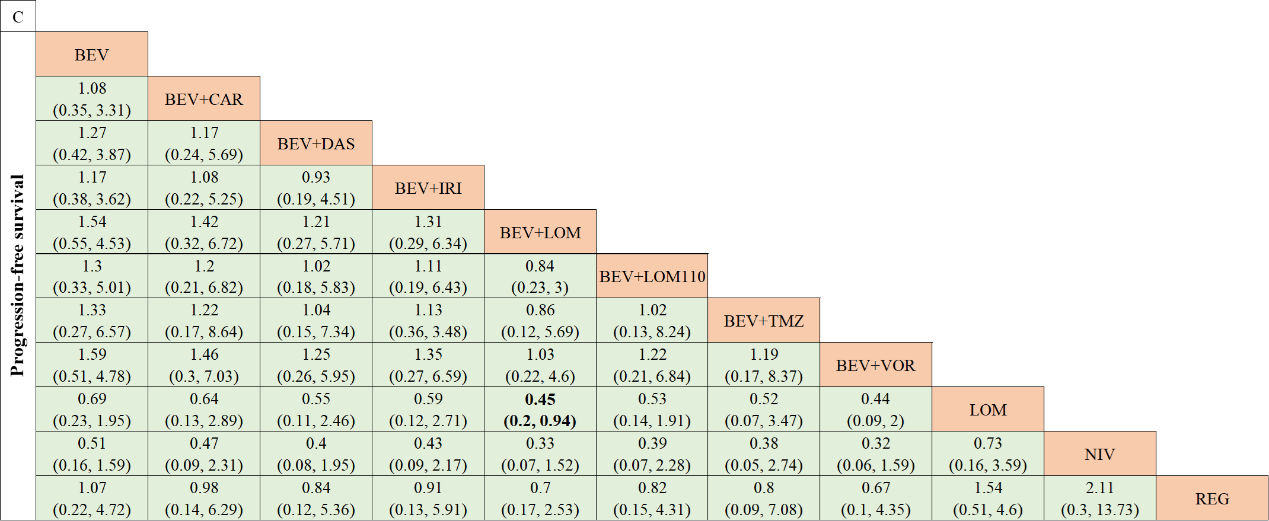


**B**


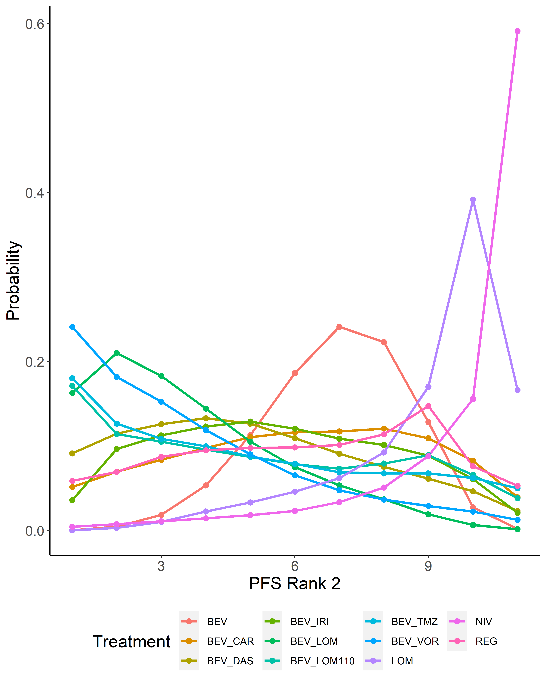


**C**


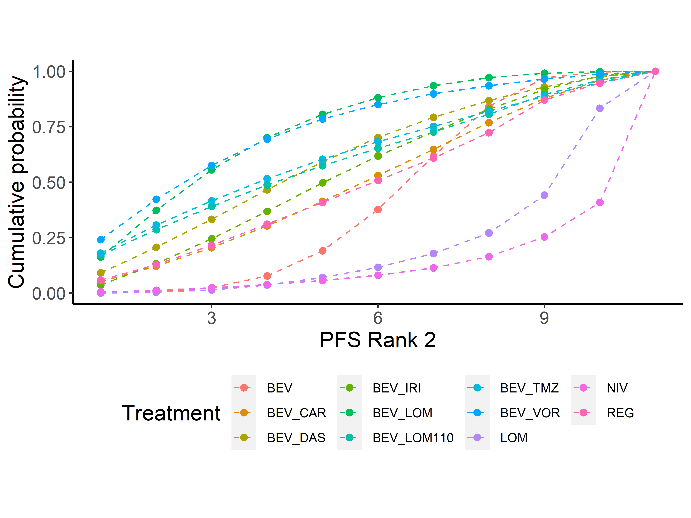


**Supplementary figure 4 Bayesian ranking results of the sensitivity analysis.** (A) Pooled HRs (95% credible intervals) for PFS in the sensitivity analysis. (B) Bayesian ranking profiles of comparable treatments on PFS. (C) SUCRA ranking of comparable treatments on PFS. BEV = bevacizumab; CAR = carboplatin; DAS = dasatinib; IRI = irinotecan; LOM = lomustine (90mg/m^2^); LOM110 = lomustine (110mg/m^2^); TMZ = temozolomide; VOR = vorinostat; NIV = nivolumab; REG = regorafenib.
